# Modeling Alzheimer’s disease with brain organoids: mechanisms, applications, and future directions

**DOI:** 10.3389/fcell.2026.1791272

**Published:** 2026-07-08

**Authors:** Qing Zhao, Songtao Li, Yanxiu Ju, Xiangyi Kong, Xilin Liu

**Affiliations:** 1 Department of Neurology, China-Japan Union Hospital of Jilin University, Changchun, China; 2 Engineering Laboratory of Memory and Cognitive Impairment Disease of Jilin Province, China-Japan Union Hospital of Jilin University, Changchun, China; 3 Department of Vascular Surgery, China-Japan Union Hospital of Jilin University, Changchun, China; 4 Department of Hand and Foot Surgery, China-Japan Union Hospital of Jilin University, Changchun, China

**Keywords:** 3D cell culture, Alzheimer’s disease, brain organoids, disease modeling, drug screening, induced pluripotent stem cells, neurodegenerative diseases, precision medicine

## Abstract

Human pluripotent stem cell-derived brain organoids have emerged as a transformative platform for modeling Alzheimer’s disease (AD), thus addressing long-standing translational obstacles posed by the disease’s complex etiology and interspecies differences. This review systematically examines methodological advances in brain organoid technology, from basic fabrication and brain-region-specific organoids to multicellular assembloids that incorporate microglia and vascular components, with an emphasis on strategies for overcoming fetal-like phenotypes. We surveyed literature published between 2018 and April 2026 that focused on human iPSC-derived organoid models that recapitulate core AD pathologies, including Aβ plaques, tau tangles, neuroinflammation, and blood–brain barrier dysfunction. Key findings demonstrate that organoids effectively capture genotype–phenotype relationships for major AD genes (*APP*, *PSEN1*, *PSEN2*, and *APOEε4*), enable the dissection of signaling pathway dysregulation (Wnt/β-catenin), and when combined with CRISPR editing and single-cell multi-omics, reveal cell-type-specific disease mechanisms. Organoids have also been successfully applied to patient-specific “avatar” models and high-throughput drug screening, thus advancing precision medicine approaches. However, current technological bottlenecks—including a lack of functional vascularization, batch-to-batch variability, and insufficient standardization—limit the full recapitulation of chronic, age-dependent AD pathology. This review critically evaluates these limitations, addresses ethical considerations surrounding neural organoids, and looks forward to future integration with artificial intelligence, spatial omics, and multi-organ systems to accelerate the translation of organoid-based discoveries into clinical applications.

## 1 Introduction

Alzheimer’s disease (AD) is a progressive neurodegenerative disorder characterized by cognitive decline, memory impairment, and behavioral disturbance, with defining neuropathological features including extracellular amyloid-beta (Aβ) plaques and intracellular neurofibrillary tangles composed of hyperphosphorylated tau. As global life expectancy increases, AD has emerged as a major public health challenge, with substantial healthcare, economic, and societal consequences (Rojas-Gualdrón et al., 2024; Guo et al., 2025; Yu D.-T. et al., 2025). The COVID-19 pandemic has compounded this burden, as SARS-CoV-2 infection may accelerate AD progression through enhanced neuroinflammation and oxidative stress while simultaneously disrupting healthcare access and psychosocial support for vulnerable populations (Iwatsubo et al., 2023; Paschalidis et al., 2023). Recent regulatory approval of anti-Aβ monoclonal antibodies, including lecanemab, marks the first demonstration of disease-modifying effects in clinical trials; however, their high cost and limited accessibility raise ethical and economic concerns, particularly in low- and middle-income countries (DePew, 2022; Rubin, 2022; Frisoni et al., 2025a). Their limited accessibility in such countries further exacerbates global inequities in AD treatment (Rubin, 2022; Frisoni et al., 2025b). The multifactorial etiology of AD, involving complex interactions among genetic, environmental, and metabolic factors, continues to impede the development of effective and broadly accessible therapies.

Traditional experimental systems, including two-dimensional (2D) cell cultures and transgenic animal models, have provided foundational insights but remain constrained by limited translational relevance. Although 2D cultures enable high-throughput screening, they fail to replicate the three-dimensional architecture and multicellular interactions of the human brain, resulting in oversimplified modeling of Aβ aggregation and tau hyperphosphorylation (Guido et al., 2023; Prajapat et al., 2023). Moreover, these systems lack critical cellular components of the AD microenvironment, such as astrocytes, microglia, and vascular cells, which actively regulate neuroinflammation, Aβ clearance, and synaptic homeostasis (Prasannan et al., 2022; Kim et al., 2025). The use of immortalized cell lines and genetically modified systems that overexpress pathogenic proteins further obscures early disease mechanisms and introduces non-physiological conditions (Prasannan et al., 2022; Valmiki et al., 2025). Although transgenic mouse models have been instrumental, species-specific differences in glial biology, aging processes, and protein aggregation limit their ability to fully recapitulate human AD pathology, particularly neuronal loss and tauopathy (Souder et al., 2021; De and Singh, 2022). Repeated failures of Aβ-targeted therapies in clinical trials underscore the need for human-relevant models that better capture disease complexity and predict therapeutic efficacy (Chen X. et al., 2021; Souder et al., 2021; Volloch and Rits-Volloch, 2024). The development of human-induced pluripotent stem cell (iPSC)-derived brain organoids has introduced a transformative platform that addresses many limitations of conventional models. These three-dimensional (3D), self-organizing structures recapitulate key aspects of human neurodevelopment, including cellular diversity, regional specification, and complex cell–cell interactions while preserving the donor’s genetic background (Jacob et al., 2021; Chai et al., 2023). Organoid systems have revealed AD-associated phenotypes not previously observed in traditional experimental models. The exposure of iPSC-derived brain organoids to human serum, modeling blood–brain barrier (BBB) disruption, induces Aβ aggregation, tau phosphorylation, and synaptic dysfunction, thereby recapitulating key features of sporadic AD (Chen X. et al., 2021). Patient-derived organoids harboring familial AD mutations have enabled high-resolution genotype–phenotype analyses. APOE ε4 organoids exhibit accelerated Aβ42 accumulation and transcriptomic profiles resembling those of AD patient brains (Kim et al., 2025). Organoids derived from patients with Dutch-type cerebral amyloid angiopathy patients (APP E693Q mutation) develop rapid Aβ40 deposition without exogenous overexpression, faithfully modeling vascular amyloid pathology (Daoutsali et al., 2023). CRISPR/Cas9-edited isogenic organoids carrying the APP V717I mutation demonstrate a downregulation of genes involved in synaptic function and axonal guidance, implicating LINGO2 as a potential disease mediator (Qu et al., 2024). Beyond amyloidogenic pathways, organoid studies have identified non-canonical roles of APP in neural development and cellular homeostasis. The reduction of APP in neural stem cells promotes neurogenesis at the expense of gliogenesis via β-catenin signaling (Coronel et al., 2023). Conversely, APP overexpression protects mitochondria from oxidative stress by activating the PI3K/Akt pathway (Cimdins et al., 2019). These findings highlight pleiotropic functions of APP not fully appreciated in animal models.

The integration of multiple cell lineages within organoid systems has further revealed intercellular interactions central to AD pathogenesis. Forebrain organoids that incorporate isogenic microglia-like cells recapitulate neuroimmune crosstalk, with microglia modulating neuroinflammation through extracellular vesicle-mediated communication (Liu C. et al., 2025). These microglia-containing organoids also promote neuronal maturation and network activity compared with neuron-only cultures (Fagerlund et al., 2021). Vascularized organoids enable the investigation of BBB dysfunction in AD. Organoids with integrated endothelial networks exhibit tight junction formation and reduced core necrosis, thus facilitating modeling of neurovascular unit pathology (Sun et al., 2022; Fumadó Navarro et al., 2025). Similarly, 3D blood–brain barrier neurosphere co-cultures demonstrate that Aβ oligomers induce endothelial barrier disruption and pericyte loss—phenotypes difficult to reproduce in 2D or animal systems (Ko et al., 2023). Combined with single-cell multi-omics and gene editing technologies, these multicellular platforms have clarified the roles of major AD gene mutations, intercellular signaling networks, and dysregulated pathways such as Wnt/β-catenin (Perez-Corredor et al., 2024). Organoids have also been applied to patient-specific “avatar” models and high-throughput drug screening platforms to advance precision medicine approaches in AD (Park et al., 2021; Ji et al., 2025).

**FIGURE 1:**
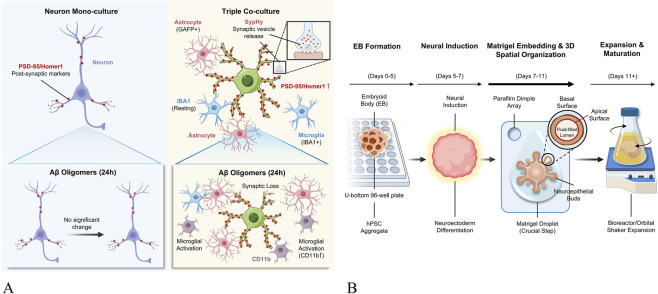
Modeling AD pathology in a triple co‐culture brain organoid system. **(A)** Neuron mono‐culture and triple co‐culture systems: neurons expressing post‐synaptic markers PSD‐95/Homer1 and synaptic vesicle protein SyPhy, co‐cultured with astrocytes (GFAP^+^) and microglia (Iba1^+^). Exposure to Aβ_1–42_ oligomers (24 h) induces microglial activation (CD11b↑) and synaptic loss. **(B)** Stepwise generation of neural organoids: human iPSC‐derived neural progenitors undergo EB formation (Days 0–5), neural induction (Days 5–7), Matrigel embedding (Days 7–11), and bioreactor expansion (Days 11+), generating neuroepithelial buds with fluid‐filled lumens. Created with BioRender.com.

Organoid technology addresses critical limitations of conventional AD research models. The multifactorial processes underlying tau pathology, Aβ deposition, and neural network dysfunction cannot be adequately captured by 2D cultures or animals (Wen et al., 2023; Rezaei et al., 2025). iPSC-derived brain organoids provide a physiologically relevant platform that preserves key human-specific biological characteristics (Chai et al., 2023). APOE ε4 organoids demonstrate accelerated Aβ accumulation and transcriptomic signatures consistent with *postmortem* AD brain tissue. Advanced modeling strategies reduce biological variability and enable the investigation of age-related influences on AD phenotypes in patient-derived organoids. These models have also clarified the contributions of glial cells and neuroinflammation to AD progression (Figure 1). Vascularized organoids and organoid-on-a-chip platforms facilitate the investigation of neurovascular interactions, BBB dysfunction, and drug delivery mechanisms in AD.

This review aims to synthesize current advances in brain organoid technology for AD research. A systematic literature review was conducted using predefined selection criteria. The search terms included “Alzheimer’s disease,” “brain organoid,” “cerebral organoid,” “3D cell culture,” and “induced pluripotent stem cells”, among related keywords. Primary emphasis was placed on studies published between 2018 and April 2026, supplemented by seminal earlier reports. The studies included utilized human PSC-derived brain organoids or assembloids for AD modeling and demonstrated key pathological features or methodological advances. Studies focused on drug development, personalized medicine, and strategies that address fetal-like organoid limitations were also included. Conference abstracts, preprints, and non-English publications were excluded. Selected studies were thematically integrated to identify convergent findings, methodological advances, and areas of controversy. Emerging trends and future directions were derived from an analysis of high-impact recent publications. As a narrative review, source selection and interpretation were guided by expert evaluation rather than formal meta-analytic methods. Readers are encouraged to consult primary studies for detailed experimental evidence.

**FIGURE 2:**
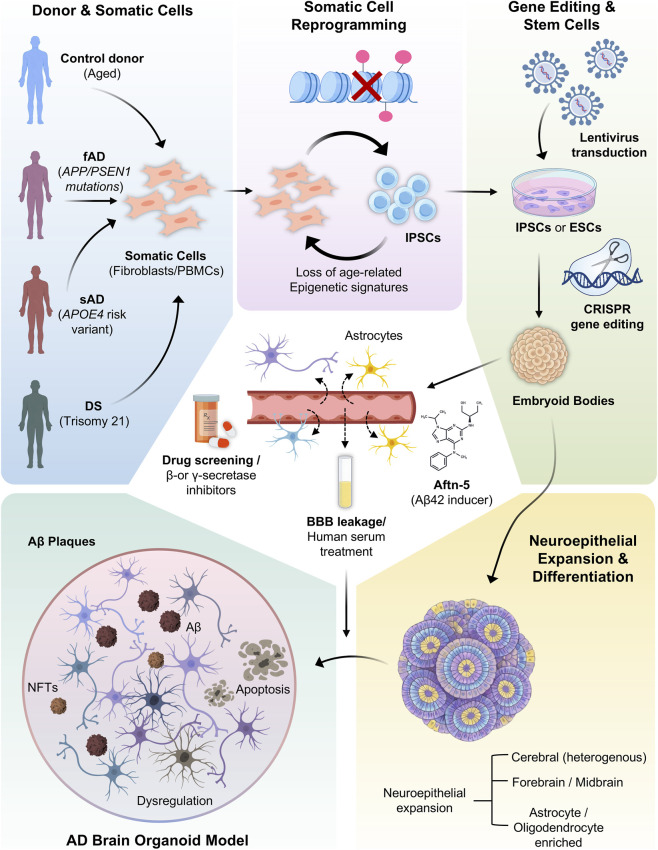
Schematic diagram illustrating the generation of brain organoid models of AD. This figure outlines the workflow for generating brain organoids from patient or control donor somatic cells, which retain the donor’s genetic background. Cell sources: somatic cells (fibroblasts or PBMCs) are obtained from control donors (aged), familial AD patients (carrying *APP* or *PSEN1* mutations), sporadic AD patients (carrying APOE4 risk variant), or Down syndrome patients (trisomy 21). Reprogramming and genetic modification: somatic cells are reprogrammed to induced pluripotent stem cells (iPSCs); during this process, most age-related epigenetic signatures are lost. Genetic modifications associated with AD (e.g., point mutations, gene knockouts) can be introduced into iPSCs or ESCs using lentiviral overexpression or CRISPR/Cas9 editing. Organoid generation and time course: genome-edited PSCs form embryoid bodies (Days 0–5), followed by neuroepithelial expansion and differentiation (Days 5–14). Organoids can be patterned into directed, brain-region-specific types (forebrain, midbrain, astrocyte-enriched, or oligodendrocyte-enriched), or maintained under unguided conditions to generate heterogeneous cerebral organoids. Cellular maturation typically requires 4–12 weeks depending on region-specific patterning. Maturation and treatment: following maturation, organoids can be treated with factors to simulate age-related events such as BBB leakage (human serum exposure) or to test drug candidates (β- or γ-secretase inhibitors). AD pathological features recapitulated: Aβ aggregates, neurofibrillary tangles (NFTs), increased apoptosis, and neuronal network dysregulation. Created with BioRender.com.

Organoid technology bridges experimental neuroscience and clinical translation by enabling disease modeling and precision therapeutic development (Figure 2). This review systematically examines methodological advances from basic organoid fabrication to complex assembloid systems. It further discusses applications in the modeling of core AD pathologies, elucidates novel mechanisms, and advances drug screening and personalized medicine. Current limitations, including incomplete vascularization, variability, and standardization challenges, are critically evaluated. Ethical considerations and future directions that integrate artificial intelligence (AI), spatial omics, and multi-organ systems are also explored. By providing a comprehensive technical and strategic framework, this review aims to accelerate the translation of organoid-based discoveries into clinical applications for AD.

## 2 Techniques for organoid synthesis

### 2.1 Self-assembly principle of brain organoids

Scientists use self-assembly principles to create embryonic brain models through the natural cell division of PSCs that form brain organoids. The process requires neural progenitors to self-organize into 3D structures that duplicate the brain development pattern of cytoarchitecture, cellular diversity, and functional brain connections (Jacob et al., 2021; Hong H. et al., 2024). Brain organoids develop through self-patterning processes which differ from traditional monolayer cultures because they respond to morphogen gradients and cell–cell interactions and biomechanical cues. The system generates synaptic networks and enables neuronal migration. This develops region-specific progenitor zones through self-patterning processes (Jacob et al., 2021; Tsai et al., 2025) which need to repeat all developmental stages that occur inside the body. The process initiates with neural induction, followed by dorsal-ventral and rostral-caudal patterning, and finishes with gliogenesis through established signaling pathways. These pathways include bone morphogenetic proteins (BMPs), sonic hedgehog (SHH) and Wingless-related integration (WNT) sites, and fibroblast growth factor (FGF) (Jacob et al., 2021; Ikeda et al., 2024). The ventral patterning process enables SHH to produce GABAergic interneurons; however, SHH follows different developmental paths in dorsal forebrain organoids, which use modified SMAD and Wnt signaling pathways to support cortical development (Sivitilli et al., 2020; Ikeda et al., 2024).

Beyond biochemical induction, spatial confinement imposed by the physical microenvironment plays a critical role in reducing inter-organoid variability. Traditional non-microwell methods (e.g., forming embryoid bodies (EBs) in culture flasks using dispase, as in the control group) yield EBs with significantly lower circularity than optimized microwell platforms. Furthermore, subsequent organoid development shows delayed surface wrinkling/folding, scarce and irregular lumen structures, and a substantially thinner mature neuronal layer. In addition, traditional Matrigel-embedding methods suffer from batch-to-batch inconsistency and long-term culture drawbacks, whereas the microwell platform presented here achieves more consistent and efficient generation of human cerebral organoids under Matrigel-free (ECM-free) conditions (Chen C. et al., 2021).

To address the limitation of heterogeneous embryoid body (EB) formation in conventional suspension cultures—which allow uncontrolled aggregate fusion and result in a wide range of sizes and shapes—engineered microwell arrays have been developed via soft lithography. Fabricated from cell-repellent poly (ethylene glycol) (PEG), these microwell platforms restrict each well to a defined geometry, forcing cells to settle into a single aggregate per well (Karp et al., 2007). An approach reduces luminal feature variability to a coefficient variation of only 5%–10% of the mean values, which is significantly lower than the estimated 30%–60% variability in traditional Matrigel-embedded methods. In addition, the microwell platform generates embryoid bodies (EBs) with significantly higher circularity and more consistent morphology than non-microwell controls (Chen C. et al., 2021).

Importantly, geometric confinement not only dictates final organoid size but also influences differentiation trajectories by modulating local cell density and associated signaling pathways. In 3D cerebral organoids—a confined microwell-like environment—higher cell density and intact cell–cell adhesion enhance Notch signaling (DLL1/3/4, NOTCH1/4, and HES5) and downregulate integrin-mediated cell–matrix adhesion compared to 2D monolayers. This altered signaling balance does not increase neural progenitor proliferation (which is actually lower in organoids) but promotes the differentiation of radial glia into intermediate progenitors and outer radial glia, thereby enhancing cortical neurogenesis, including the generation of TBR1^+^ deep-layer and CTIP2^+^ layer-5 neurons. The Hippo/YAP pathway is also differentially expressed, though its specific role remains unvalidated (Scuderi et al., 2021). Microwell-based spatial confinement serves as a simple yet powerful engineering strategy to enhance the reproducibility and maturation of brain organoids for disease modeling and drug screening.

In brain organoid development, self-assembly is orchestrated by geometric confinement together with biochemical signaling regulation—equally critical—which operates through both extrinsic morphogen gradients and intrinsic cell–cell communication. The physical constraints imposed by microwell architecture directly influence these signaling outcomes through density-dependent mechanotransduction. Human iPSC-derived telencephalic organoids (ORGs), by preserving cell–cell contacts, effectively maintain Notch signaling activity (including DLL1, NOTCH1, and HES5), thereby promoting the generation of intermediate progenitors and outer radial glia and successfully producing TBR1^+^ deep-layer and CTIP2^+^ upper-layer cortical neurons (Scuderi et al., 2021). In contrast, 2D monolayers (MONs) generated by dissociation lose cell–cell contacts, leading to the significant downregulation of Notch pathway genes, accompanied by neurogenic defects and aberrantly elevated integrin signaling. Prompt reaggregation (REAG) partially restores Notch expression and neurogenesis but fails to fully recover polarity. This study did not address YAP/TAZ. YAP is highly expressed and is present in a dephosphorylated (active) form in ferret and human basal progenitors, correlating with their high proliferative capacity; in contrast, mouse basal progenitors exhibit low YAP expression (Kostic et al., 2019). Conditional expression of constitutively active YAP in mouse basal progenitors promotes their proliferation and increases upper-layer neuron production, whereas the inhibition of YAP activity in ferret and human neocortex reduces basal progenitor abundance. YAP is both necessary and sufficient for basal progenitor proliferation and likely contributed to neocortical expansion during evolution. This study did not investigate Notch signaling or microwell cultures. Thus, physical confinement and biochemical signaling are not independent variables but are, rather, integrated inputs that collectively guide the self-assembly trajectory. Understanding this integration is essential for designing optimized culture platforms that recapitulate human brain development with high fidelity.

To overcome the batch-to-batch variability and undefined compositional complexity of animal-derived matrices such as Matrigel, researchers have developed various well-defined synthetic extracellular matrices (ECMs) and their polymer conjugates to guide self-assembly. These synthetic scaffolds incorporate specific bioactive moieties to achieve precise control over cellular behavior; this represents a critical shift from ill-defined biological substrates toward chemically defined, reproducible culture systems.

Biofunctionalized gelatin-based hydrogels have been engineered as an alternative to Matrigel by presenting specific signaling cues. For example, GelMA-Cad hydrogel—generated by covalently conjugating neural cadherin (N-cadherin) peptides to gelatin methacryloyl (GelMA)—supports cortical organoid culture in a defined, Matrigel-free environment. This peptide-functionalized matrix mimics homotypic cell–cell interactions through the presented N-cadherin motifs. Compared to Matrigel, cortical organoids cultured in GelMA-Cad hydrogels map more closely to human fetal brain populations and exhibit a higher frequency of spontaneous excitatory post-synaptic currents (sEPSCs), indicating accelerated synaptic maturation and enhanced early electrophysiological activity (Kjar et al., 2024).

Synthetic polyethylene glycol (PEG) hydrogels feature tunable mechanical stiffness, defined biochemical composition, and controlled degradability, making them attractive synthetic substrates for neural organoid assembly. By sequentially co-culturing human iPSC-derived endothelial cells, pericytes, neural progenitor cells, and microglia on the surface of PEG hydrogels in a defined temporal order, 3D planar neural organoids (PNOs) can be obtained. Compared to neural tissues assembled on Matrigel surfaces, PNOs exhibit greater neuronal diversity (encompassing various cortical markers and synaptic proteins), higher expression of neurovascular and neuroinflammatory genes, and significantly reduced batch-to-batch variability. Furthermore, when compared to traditional brain organoids grown in Matrigel suspension, PNOs also better express neural, vascular, and inflammatory genes. PNOs contain functional microglia that respond to lipopolysaccharide (LPS) stimulation by producing TNF-α and IL-6 and are sensitive to anti-inflammatory drugs (celecoxib and donepezil). Owing to their flat, semi-translucent morphology, PNOs are suitable for continuous live-cell imaging, thereby establishing them as a physiologically relevant *in vitro* model of neuroinflammation (Majumder et al., 2024).

Engineered extracellular matrix (EECM) provides structural support and authentic cell–matrix interactions for brain organoids. The EECM platform consists of a highly porous poly (lactic-co-glycolic acid) (PLGA) scaffold that supports a suspended fibrillar network of full-length human fibronectin. This interstitial matrix-mimicking system allows human pluripotent stem cells to attach, differentiate, and self-organize into brain organoids. Compared to Matrigel, EECM enhances neurogenesis, promotes glial maturation, and increases neuronal diversity. Importantly, EECM supports long-term culture for up to 7 months and generates large-volume organoids capable of producing over 250 μL of cerebrospinal fluid (CSF)-like fluid. Proteomic analysis shows that this CSF contains 280 proteins shared with adult human CSF, spanning 500 Gene Ontology pathways, with protein diversity exceeding that of previously reported brain organoid models. EECM represents a promising advanced neural engineering platform that can significantly improve the structural, cellular, and functional complexity of brain organoids (Muñiz et al., 2023; Wen et al., 2025). Collectively, these synthetic ECM platforms represent a paradigm shift from static, ill-defined culture systems to controllable, reproducible bioengineered models for AD research and drug screening.

### 2.2 Construction of brain-region-specific organoids

Scientists now use brain-region-specific organoids as a primary instrument to study human brain development and disease progression, thus enabling the modeling of conditions such as AD that affect discrete brain areas. Organoids replicating the hippocampus, cortex, and choroid plexus have been successfully generated through specific patterning cues and microenvironmental signals. Hippocampal organoid development is achieved by WNT3a supplementation, which induces ZBTB20 and KA1 expression and confers CA3 subfield identity (Ciarpella et al., 2021). Dorsal forebrain organoids enriched in excitatory neurons are generated by dual SMAD and Wnt inhibition, whereas ventral forebrain organoids enriched in inhibitory neurons require higher SHH pathway activation (Mulder et al., 2023). These region-specific models retain human-specific cytoarchitecture and cellular diversity, making them particularly valuable for studying AD mechanisms.

Recent technological advances have improved the reproducibility and functionality of these organoids. Single-rosette-based methods reduce batch-to-batch variability, yielding uniform organoid size and proper laminar organization, which allows precise investigation of AD-related cortical layering defects (Jalilian and Shin, 2023). Co-culture systems that incorporate meningeal cells enhance laminar arrangement and upregulate cortical layer-specific markers, recapitulating the niche required for synaptic refinement and glial development (Tanaka et al., 2020). Microfluidic platforms further enable the assembly of multi-region assembloids, facilitating the study of long-range connectivity deficits in AD (Chen J. et al., 2023). These assembloids exhibit interregional axonal projections and functional synaptic activity, as demonstrated by calcium imaging and MEA recordings (Tsai et al., 2025).

The ability of these organoids to develop disease-relevant phenotypes makes them suitable for AD research. Choroid plexus organoids, generated using CHIR and BMP4, replicate cerebrospinal fluid secretion and barrier properties, enabling studies of Aβ clearance dysfunction (Pellegrini et al., 2020). Transcriptome analysis of patient-derived cortical and hippocampal organoids reveals dysregulated pathways involved in calcium signaling and mitophagy, correlating with neurodegenerative changes (Giandomenico et al., 2021). Functional assays using patch-clamp electrophysiology demonstrate that AD organoids develop hyperexcitability, mirroring early network dysfunction observed in patient brains (Zhang et al., 2023).

Despite these advances, challenges remain in achieving complete vascularization and full tissue maturation. Slice culture methods improve neuronal survival and axonal growth but cannot replace the need for vascularized organoids to study BBB disruption in AD (Fan et al., 2024). Emerging strategies, including endothelial cell co-culture and rodent transplantation, are being developed to address this limitation (Pavon et al., 2024). Future research will focus on generating assembloids that incorporate multiple glial cell types and employ CRISPR-Cas9 editing of AD risk genes for personalized therapeutic evaluation (Lei et al., 2024). Combined with multi-omics and high-throughput imaging, brain-region-specific organoids will continue to advance targeted therapeutic development for AD (Zhu et al., 2023).

### 2.3 Complex system construction: assembloids and co-culture

As an extension of self-assembly principles, the fusion of different brain region-specific organoids generates “assembloids.” By fusing dorsal cortical and ventral forebrain organoids, functional glutamatergic-GABAergic circuits can be constructed, enabling the study of neurodevelopmental disorders such as Timothy syndrome. Assembloids derived from different brain regions enable the reconstruction of neural circuits to study tau propagation between brain areas. By fusing specific organoid types, such as cortical and spinal spheroids, scientists can model white-matter pathways at macroscale (Revah et al., 2022; Osaki et al., 2024). These connected assembloids exhibit improved oscillatory performance and complex functions, allowing the investigation of intricate neural systems (Osaki et al., 2024). Specifically, 3D neuron-astrocyte co-cultures derived from hiPSCs facilitate the study of spontaneous tau aggregation without exogenous seeds while minimizing non-cell-autonomous effects encountered in conventional seeding models (Batenburg et al., 2023). This co-culture system maintains synaptic connections and spontaneous calcium signals for up to 8 weeks within extracellular matrix scaffolds, providing a physiologically relevant environment for studying tau-related disease progression.

The modular nature of assembloids enables precise control over cellular composition and connectivity. Assembloids combining excitatory cortical neurons and inhibitory interneurons model epileptiform activity and elucidate how interregional axonal bundles act as hubs for network failure synchronization (Saberi et al., 2022; Pan et al., 2024). Cortico-motor assembloids, which unite cortical, spinal, and muscular elements to create functional motor systems, allow the investigation of tau-induced synaptic deterioration (Andersen et al., 2020). These systems integrate seamlessly with optogenetic stimulation, calcium imaging, and single-cell omics, enabling mechanistic studies (Andersen et al., 2020; Pan et al., 2024). Microfluidic devices have been used to build entorhinal-hippocampal circuits, allowing the real-time investigation of tau propagation and its effects on network function (Hanssen et al., 2024). Tesla-valve-inspired microtunnels guide axonal growth and produce nerve projections that recapitulate natural tissue patterns, thus enabling studies of tau transmission mechanisms relevant to AD brain networks.

This approach has applications in drug discovery and personalized therapy. Isogenic organoid lines generated by episomal vector insertion allow the identification of the cell-specific effects of tau mutations (Nassor et al., 2020). These models have been used to evaluate therapeutic candidates, including PIKFYVE kinase inhibitors that reduce glutamate toxicity in tau-mutant organoid cultures (Bowles et al., 2021). The scalability of the assembloid system in 96-well plate formats with high-throughput imaging enables large-scale compound screening for tau aggregation inhibitors and synaptic protectants (Saberi et al., 2022; Batenburg et al., 2023). By replicating tau transmission between brain regions and incorporating advanced functional readouts, assembloid technology serves as a critical tool for understanding neurodegenerative disease mechanisms and developing novel therapeutics.

### 2.4 Introduction of multiple cell lineages: microglia and vascular endothelial cells strategies

**FIGURE 3:**
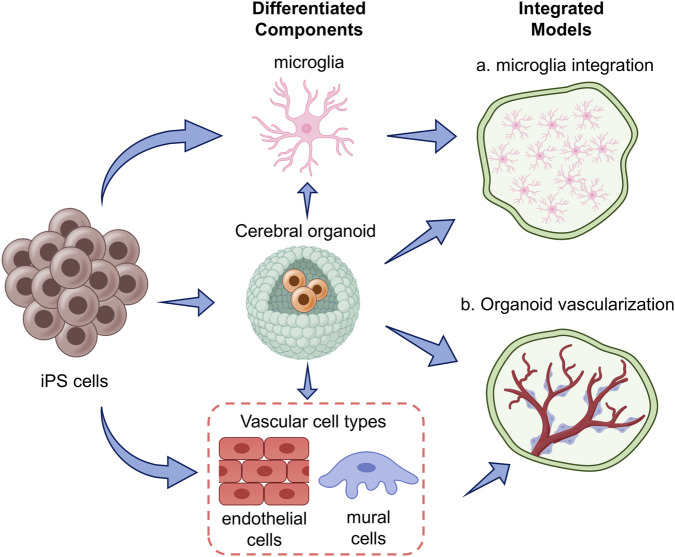
Incorporating non-neuronal cell types into brain organoids. This schematic illustrates strategies to improve the physiological relevance of cerebral organoids by incorporating additional cell types that do not develop endogenously within standard organoid protocols. iPSC-derived microglia are added to cerebral organoids to model neuroimmune interactions that underlie healthy brain function and neurodegeneration. Microglia can be derived from the same iPSC line (isogenic) or from a different donor line, allowing studies of microglial–neuronal crosstalk, synaptic pruning, and neuroinflammatory responses in AD. iPSC-derived cerebrovascular endothelial cells and mural cells (pericytes, smooth muscle cells) are incorporated into organoids to promote vascular network formation, enhance cell viability by reducing hypoxic core necrosis, and enable studies of neurovascular interactions and BBB function. Cell sources: human iPSCs (from control donors, fAD patients with APP/PSEN1/PSEN2 mutations, sAD patients with APOE4 risk variant, or Down syndrome patients). Organoid types: cerebral organoids with integrated microglia or vascularized cerebral organoids. Time points: microglia are typically added during organoid maturation (approximately Days 40–60 of differentiation); vascular cells are incorporated at early stages (Days 10–20) to promote network formation. Maturation for functional studies (microglial ramification, endothelial tight junction formation) requires an additional 2–4 weeks. Created with BioRender.com.

The combination of microglia and vascular endothelial cells with assembloids allows scientists to conduct groundbreaking simulations to model intricate brain development processes and disease mechanisms by studying neuronal and immunological and vascular system interactions (Figure 3). Organoids from different cell types are combined to create assembloids to investigate essential multicellular interactions of neurovascular unit functions and neuroimmune system communication (Bartalska et al., 2022; Sun et al., 2022; Onesto et al., 2025). The brain contains microglia that develop from yolk sac macrophages during embryonic development to become its native immune cells. These cells control neurogenesis and synaptic pruning and circuit development (Shiraki et al., 2022; Dowbaj et al., 2025). Traditional methods of cerebral organoid development fail to produce microglia because these cells derive from mesodermal tissues. Therefore, researchers must use specific cell differentiation methods or culture techniques to add these cells to the system (Bartalska et al., 2022; Sun et al., 2022). Similarly, the absence of vascular endothelial cells (ECs) in traditional neural organoids restricts the study of neurovascular interactions, despite the fact that ECs are crucial for creating the BBB and giving neural tissues trophic support (Payne et al., 2022; Sun et al., 2022).

The lack of suitable models for these studies is addressed through new assembloid systems, which unite vascular organoids (VOs) with human iPSC-derived microglia-like cells and neural organoids in the study of human-specific immune surveillance and BBB development and angiogenesis (Sun et al., 2022; Hong et al., 2025; Onesto et al., 2025). Fused vascularized brain assembloids contain functional neurovascular units to show ECs expressing CLDN5 and ZO-1 tight junction proteins for BBB-like functions and microglia cells that respond to immunological triggers (Payne et al., 2022; Sun et al., 2022). The models show that vascular networks form through biological mechanisms that defend brain progenitor cells from death while simultaneously increasing survival chances (Sun et al., 2022; Dowbaj et al., 2025). Scientists can now study how microglia perform two separate functions: synaptic remodeling and their role in neuroinflammatory and neurodegenerative diseases (Bartalska et al., 2022; Shiraki et al., 2022; Onesto et al., 2025).

Regarding technology, the generation of microglia-like cells has been made possible by the use of BMP4 in differentiation protocols, while the derivation of ECs for vascularized assembloids is made possible by temporary mesoderm induction (Bartalska et al., 2022; Payne et al., 2022; Sun et al., 2022). Obtaining active blood flow and guaranteeing long-term stability are persistent obstacles; however, incorporating microfluidic devices or *in vivo* grafting could assist in overcoming these limitations (Sun et al., 2022; Li X. et al., 2024). The assembloid platform shows promise for individualized medical treatment because researchers can study leukoencephalopathies and retinal degeneration through patient-derived cells, which develop these conditions because of microglial cell malfunction (Bartalska et al., 2022; Shiraki et al., 2022; Dowbaj et al., 2025).

Multicellular assembloids unite neuroscience with immunology and vascular biology research (Yang et al., 2024; Hong et al., 2025; Onesto et al., 2025). Improving spatial organization, adding more cell types, and using CRISPR screens to find new regulators of microglial-EC-neural connections are also future possibilities (Hong et al., 2025; Onesto et al., 2025). Scientists can use assembloids to study human brain development and speed up neurological disease treatment development through these research methods.

## 3 Overcoming the core challenges in AD modeling

### 3.1 Challenges in organoid maturation

The development of human neurodevelopment through iPSC-derived brain organoids has made substantial progress; however, these models do not perfectly duplicate the epigenetic patterns that exist in embryonic brain tissue. The main difficulty in working with iPSCs stems from their complex composition of different cell types and their incomplete reprogramming process, which maintains somatic cell epigenetic information and creates abnormal DNA methylation patterns (Rouhani et al., 2022; Czerminski et al., 2023). The unstable epigenetic state leads to organoid development that differs from fetal brain development because the cells follow different paths of differentiation (Rouhani et al., 2022; Czerminski et al., 2023). The process of organoid maturation needs long-term cell culture, resulting in the development of somatic mutations and specific genetic changes that indicate possible changes in epigenetic markers (Rouhani et al., 2022; Czerminski et al., 2023). Organoids fail to achieve their native chromatin states and three-dimensional genomic organization because they lack essential biological signals that exist during *in vivo* development. The absence of vascularization and immunological contacts and specific mechanical forces that occur *in vivo* restricts organoids’ ability to develop native chromatin states and 3D genomic architecture (Chiaradia et al., 2023; Ferraro et al., 2025; Velazquez Ojeda et al., 2025).

Organoids develop laminar cortical structures; however, the production of intermediate progenitor cells and upper-layer neurons remains lower than fetal levels due to a failure to duplicate proper embryonic gene expression patterns (Chiaradia et al., 2023; Frenz-Wiessner et al., 2024). The problems become more severe because of the inconsistent extracellular matrix composition between batches, which creates unmanageable environmental signals that might alter how cells develop their epigenetic patterns (Ferraro et al., 2025; Velazquez Ojeda et al., 2025). New single-cell RNA sequencing studies show that organoids of identical appearance contain different gene expression profiles because of epigenetic variations which hide important disease-related characteristics in Down syndrome models of neurodevelopmental disorders (Chiaradia et al., 2023; Czerminski et al., 2023). Wnt signaling serves as a vital pathway for fetal brain development; however, researchers activate this pathway during hypoxia-mimicking cultures to boost neurogenic potential. Laboratory cultures have failed to duplicate environmental factors that affect fetal epigenetic development during pregnancy (Frenz-Wiessner et al., 2024; Velazquez Ojeda et al., 2025). Present techniques need sophisticated bioengineering to develop precise fetal brain development models by using synthetic ECM scaffolds, which can be made with different stiffness levels and microfluidic systems that mimic blood vessel networks (Chiaradia et al., 2023; Ferraro et al., 2025). To separate technical artifacts from physiologically significant epigenetic alterations in organoid models, future studies must also give priority to established techniques and isogenic controls (Chiaradia et al., 2023; Czerminski et al., 2023).

### 3.2 Aging induction strategies

Modeling late-onset neurodegenerative diseases such as AD requires recapitulating age-related cellular phenotypes because AD pathology is strongly influenced by aging processes that are not inherently present in standard iPSC-derived organoids. To address this limitation, three main strategies have been developed to induce aging-associated features in human cellular models: (i) genetic methods, (ii) chemical treatments, and (iii) direct reprogramming that preserves donor age-related epigenetic marks. Each approach has distinct mechanisms, advantages, and limitations.

#### 3.2.1 Genetic method

The genetic method induces cellular aging and age-related disease phenotypes by forcing the expression of progerin, a truncated abnormal version of lamin A. Progerin accumulation disrupts nuclear structure, chromatin organization, and DNA stability, recapitulating features of Hutchinson–Gilford progeria syndrome (HGPS) and accelerating aging in culture (Chojnowski et al., 2020; Benedicto et al., 2024). Mechanistically, progerin causes replication stress by blocking DNA replication forks, leading to DNA degradation through nuclease activity and impairing RAD51 repair factor binding. These events trigger persistent DNA damage and activate the cGAS/STING/IFN inflammatory pathway (Coll-Bonfill et al., 2020; Joudeh et al., 2024). Progerin also promotes stress-induced premature senescence (SIPS) via p53-dependent pathways (Xiang et al., 2022).

The progerin-based system is useful for studying both HGPS and natural aging mechanisms, as it recapitulates nuclear blebbing, heterochromatin loss, and defective homologous recombination repair (Joudeh et al., 2023; Benedicto et al., 2024). However, progerin overexpression does not fully replicate all aspects of natural aging and shows context-dependent effects across cell types (Hu et al., 2020). Nevertheless, it has enabled the identification of senotherapeutic compounds such as calcitriol, which stabilizes RAD51 and reduces the cGAS/STING/IFN response (Coll-Bonfill et al., 2020) as well as clarifying cell-type-specific contributions to vascular aging (Benedicto et al., 2024).

#### 3.2.2 Chemical method

Chemical interventions use telomerase inhibitors or mitochondrial stressors to induce senescence-like states. For example, KML001 (sodium meta-arsenite) disrupts telomere protection, causing mitochondrial damage and p53-dependent senescence (Borghini et al., 2024). Hydrogen peroxide (H_2_O_2_) induces oxidative damage preferentially at telomeric repeats, leading to accelerated telomere shortening and chromosomal instability (Borghini et al., 2024).

The chemical approach enables multiplexed targeting and high-throughput experiments. However, small-molecule cocktails often cannot fully replicate the complexity of aging because they may block only selected aging pathways (Armanville et al., 2025). Despite this limitation, chemical induction has been used to study age-related diseases and discover senolytic agents, such as those targeting ER-mitochondria calcium flux (Puebla-Huerta et al., 2025). Nanoparticle-based delivery systems are being developed to improve specificity and reduce off-target effects (Akgun, 2025; Armanville et al., 2025).

#### 3.2.3 Direct reprogramming

Direct reprogramming converts somatic cells into induced neurons (iNs) or human induced dorsal forebrain precursor (hiDFP) cells without passing through a pluripotent state, thereby preserving donor age-related epigenetic and metabolic signatures, including DNA methylation patterns, telomere length, and senescence-associated gene expression (Zhang and Gladyshev, 2020; McCaughey-Chapman et al., 2023). For instance, hiDFPs derived from aged donors maintain high CDKN2A expression (20- to 40-fold higher than in iPSCs) and exhibit mitochondrial dysfunction and elevated ROS production, making them suitable for modeling age-dependent neurodegenerative diseases such as AD (Jiang et al., 2021; McCaughey-Chapman et al., 2023).

This approach avoids the need for artificial aging induction and preserves the natural aging environment. Directly reprogrammed neurons maintain age-dependent neurite outgrowth patterns and synaptic architecture (Berdugo-Vega et al., 2020). However, challenges such as limited cell survival and heterogeneity persist. Advances in region-specific transcription factors, G1-phase synchronization, and antisense oligonucleotide (ASO)-mediated PTB suppression are improving the precision and scalability of direct reprogramming (Maimon et al., 2021; Kraskovskaya et al., 2023). This technology holds promise for both disease modeling and regenerative therapies, as it avoids teratoma formation associated with iPSCs (Antón-Fernández et al., 2024; Ye, 2025).

The three aging induction strategies—genetic, chemical, and direct reprogramming—offer complementary tools for introducing age-related features into AD organoid models. Their appropriate selection depends on the experimental question, desired throughput, and need to preserve donor-specific aging signatures.

## 4 Modeling core AD pathologies with brain organoids

### 4.1 Extracellular deposition and aggregation of amyloid-β plaques

Organoid technology has brought a new level of research capability to AD studies because it allows the investigation of core disease mechanisms that involve Aβ plaque development and Aβ accumulation in cellular membranes (Table 1). The 3D organoid system provides a biological environment that duplicates brain tissue conditions in the study of Aβ plaque development and its resulting effects; it provides a more accurate platform for studying Aβ plaque development and its subsequent effects because it duplicates the human brain environment that cannot be matched by 2D cell cultures (Prasannan et al., 2022; Whitehouse et al., 2025).

**TABLE 1:** Summary of key brain organoid studies in Alzheimer’s disease research.

Study	Organoid model	Culture duration	Key AD-related finding	Novel insights beyond traditional models
Chen et al. (2021b)	iPSC-derived brain organoids exposed to human serum	Up to 150 days	Aβ aggregation, tau phosphorylation, synaptic dysfunction; recapitulation of sporadic AD features	First model to capture sporadic AD multifactorial nature without genetic mutations; demonstrates that BBB leakage alone can induce AD pathology
Kim et al. (2024)	Directly reprogrammed 3D brain organoids from APOE ε4 fibroblasts	60–90 days	Accelerated Aβ42 accumulation; gene expression profiles matching AD patient brains	Preserves age-related epigenetic marks; shows APOE ε4 organoids develop AD phenotypes faster than isogenic controls
Daoutsali et al. (2023)	Cerebral organoids from Dutch-type CAA patients (*APP* E693Q mutation)	52–90 days	Rapid Aβ40 deposition; vascular amyloid pathology; disrupted TGF-β signaling	Recapitulates vascular amyloid pathology without exogenous Aβ overexpression; demonstrates faster aggregation than conventional AD models
Qu et al. (2024)	CRISPR/Cas9-edited isogenic organoids (*APP* V717I mutation)	120 days (including 90 days post-xenograft)	Downregulation of synaptic and axonal guidance genes; LINGO2 identified as disease mediator	Single-nucleus RNA sequencing of xenografted neurons reveals cell-type-specific vulnerabilities; identifies novel signaling pathways
Liu et al. (2025a)	Forebrain organoids with isogenic microglia-like cells	100 days	Microglia modulate neuroinflammation via extracellular vesicle-mediated communication; differential gene expression in response to AD EVs	Demonstrates isogenic neuroimmune crosstalk in 3D system; reveals EV-based microglial regulation of inflammation
Fagerlund et al. (2021)	Cerebral organoids integrated with microglia-like cells	60–90 days	Microglia promote neuronal maturation, synaptic function, and network activity; increased gamma-band oscillations	Shows that microglia are essential for proper neuronal network development in organoids, not observed in neuron-only cultures
Sun et al. (2022)	Vascularized brain organoids with endothelial networks	30–60 days	Formation of functional neurovascular units; reduced core necrosis; expression of tight junction proteins	First demonstration of self-organizing vascular networks within brain organoids; enables study of neurovascular interactions
Fumadó Navarro et al. (2025)	Cerebral organoids with integrated endothelial networks	Up to 60 days	Enhanced neuronal maturation; reduced apoptosis; improved electrophysiological function	Demonstrates that vascularization mitigates core necrosis and supports long-term organoid health
Ko et al. (2023)	3D blood–brain barrier neurosphere co-culture model	7–21 days	Aβ oligomers induce endothelial barrier disruption and pericyte loss; elevated soluble Aβ40 levels.	Recapitulates Aβ-induced vascular damage in controlled 3D system; enables real-time monitoring of BBB integrity
Coronel et al. (2023)	Human neural stem cells (complementary to organoid studies)	7–14 days differentiation	APP reduction shifts differentiation toward neurogenesis and decreases gliogenesis via β-catenin signaling	Reveals non-amyloidogenic role of APP in neural fate determination; not previously appreciated in animal models
Cimdins et al. (2019)	Neuronal models (complementary to organoid studies)	Not specified	APP overexpression protects mitochondria from oxidative damage through PI3K/Akt pathway activation	Demonstrates neuroprotective function of APP independent of amyloid pathology
Perez-Corredor et al. (2024)	iPSC-derived cerebral organoids from PSEN1 E280A carriers with APOE3 Christchurch variant	60–90 days	APOE3Ch enhances Wnt/β-catenin signaling; reduces tau phosphorylation; protects against PSEN1 mutation-induced pathology	Reveals protective mechanism of APOE3Ch variant in human 3D context; identifies Wnt pathway as therapeutic target
Ji et al. (2025)	Vascularized neuroimmune organoids exposed to sporadic AD brain extracts	4 weeks post-exposure	Development of tau tangles, Aβ plaques, neuroinflammation, and synapse loss	First model to show that sporadic AD brain extracts alone can induce multiple pathologies in vascularized neuroimmune organoids
Park et al. (2021)	3D brain spheroids from AD patient iPSCs	50–70 days	Aβ accumulation; caspase-induced cell death; platform for drug screening	Enables logical network-based drug screening; identifies patient-specific drug responses
Zhao et al. (2025)	Review of organoid technology advances	N/A	Summarizes key developments and optimization strategies for neurodegenerative disease modeling	Provides comprehensive overview of technological iterations and future directions

Human fibroblast-derived 3D-cultured generated brain organoids show strong tau pathology and Aβ aggregation, and APOE ε4-expressing organoids have much higher quantities of Aβ42 protein than their 2D counterparts (Kim et al., 2024). The formation of a brain-like structure in 3D occurs when Matrigel contains its ECM components laminin and collagen, supporting neuronal development and synaptic formation and resulting in improved pathological representation (Massimi et al., 2020; Prasannan et al., 2022). AD-related symptoms are enhanced by 3D architecture because 3D organoids develop Aβ plaque formation and tau hyperphosphorylation at faster rates than control samples. Furthermore, thioflavin T-positive aggregates and phosphorylated tau (p-Tau) deposits exist together with neuronal markers in the same areas of the tissue (Kim et al., 2024; Korde and Humpel, 2024).

The physical limits of 3D environments allow the investigation of Aβ plaque behavior by analyzing the geometric shape and the relationships with brain tissue and the spatial arrangement. APP/PS1 transgenic mouse cerebellum using synchrotron X-ray phase contrast tomography demonstrates that Aβ plaques in 3D organoids develop into elongated shapes that follow the same patterns as human AD brain tissue (Massimi et al., 2020). The structural simplicity of 2D cultures prevents the modeling of plaque-tissue interactions because they do not possess the required spatial organization. Three-dimensional organoids enable the study of plaque formation and the cause of neuritic dystrophy and synaptic degeneration through pre- and post-synaptic marker co-localization and the progressive reduction of synaptic puncta density (Guido et al., 2023; Kim et al., 2024). The 3D system elucidates Aβ aggregation together with cellular stress responses, including oxidative stress and mitochondrial dysfunction, at higher complexity than 2D cultures because 3D cultures need more power to function while their physical boundaries exist (Park et al., 2023; Whitehouse et al., 2025).

The 3D organoid system offers new solutions because it allows the use of patient-specific genetic data and enables the study of different cell types to addresses key restrictions found in conventional 2D cell culture models. Aβ accumulation and caspase-induced cell death is shown in 3D brain spheroids derived from iPSCs of AD patients, enabling the conduct of large-scale drug tests for neuroprotective compounds, including curcumin and nordihydroguaiaretic acid (Park et al., 2023). The tri-culture system, consisting of neurons, astrocytes, and microglia cultured on 3D microfluidic platforms, duplicates Aβ clearance mechanisms and neuroinflammatory responses to link laboratory-based studies with animal-based investigations (Garreta et al., 2021; Prasannan et al., 2022). Organoids demonstrate potential for individualized medical care because scientists have developed methods to create organoids that duplicate individual patient characteristics to improve both therapeutic options and predictive drug response models (Fan et al., 2025; Verstegen et al., 2025).

However, some challenges remain, such as the requirement to standardize organoid procedures to increase reproducibility and reduce diversity in cell type representation (Hofer and Lutolf, 2021; Kim et al., 2024). Future bioengineering techniques may enhance organoid development through their ability to create ECM-mimetic scaffolds and 3D bioprinted structures to produce better organoids and functional organ connections (Hofer and Lutolf, 2021; Zeng et al., 2024). Organoids can be combined with advanced imaging methods and computational modeling to discover new processes whereby Aβ plaques spread and cause damage to cells (Massimi et al., 2020; Zhang et al., 2022). In addition, 3D organoids serve as research tools which allow scientists to study AD because they elucidate Aβ disease mechanisms, which may lead to the development of specific treatment methods. The models serve as vital research instruments for neurodegenerative disease studies because they replicate all biological and physical components that exist in the human brain.

### 4.2 Tau tangles and trans-synaptic propagation

Organoids serve as valuable tools to study AD complex disease mechanisms because they enable researchers to create models that accurately demonstrate tauopathy and its ability to spread between neurons. Research into tau disease has expanded due to developments in 3D neuronal circuit models such as assembloids. These systems use neurons and astrocytes derived from human iPSCs to recreate the brain environment, enabling tau proteins to spread through neural pathways (Batenburg et al., 2023; Korde and Humpel, 2024). Pathogenic tau mutations are utilized to create a special 3D neuron–astrocyte co-culture system that show the first signs of tau disease inside neurons and demonstrate tau protein clustering that does not require any external seeds. The drug screening model is effective for medium-throughput applications because it works with 96-well plates and high-content imaging systems (Batenburg et al., 2023).

The assembloid platform reveals how neural circuits enable tau spread, which follows the same pattern as Braak staging observed in AD patient brains. Microfluidic devices show that tau aggregates spread between neurons through their connected axons (Katsikoudi et al., 2020; Korde and Humpel, 2024). The observed pattern of tau spread from the entorhinal cortex to hippocampal and neocortical regions follows the same pattern (Schrank et al., 2020; Lee et al., 2022). The system design includes modular components to investigate microglia cells that play a key role in tau protein spread while examining the neuroinflammation effects on the disease process (Anand et al., 2022; Batenburg et al., 2023).

The Nexopathy *in silico* (Nexis) model studies the effect of microglial cells on tau protein spread by analyzing cellular architecture. The model demonstrates that Trem2 functions as the main gene that increases AD risk by decreasing tau protein levels in specific brain areas but also speeds up tau protein spread between brain regions. The brain contains two opposing microglial cell functions which function as waste removal agents and transmit tau protein between different brain areas (Anand et al., 2022).

Recent technological developments have helped organoid engineering solve some long-existing problems with conventional models. The tripartite synapse architecture is preserved in 3D assembloids, in contrast to 2D cultures or transgenic animals, allowing for the investigation of neuron–astrocyte interactions in tau pathogenesis. The models feature astrocytes that resemble their natural structure in living tissue while they work with neurons to control tau protein removal and brain cell connection adjustments (Batenburg et al., 2023; Guido et al., 2023). Organoid culture benefits from defined animal-free matrices, in which synthetic hydrogels function with integrin-activating domains to improve reproducibility in translational research (Wijnakker et al., 2025). Single-cell transcriptomic atlases have been used to compare brain organoid development with human fetal brain stages, revealing both conserved and protocol-specific gene expression programs that guide neuronal specification, differentiation trajectories, and the emergence of functional pathways such as axon guidance, synaptic transmission, and action potential. Common cell repertoires have been identified in the integrated analyses of multiple human cortical organoid protocols against fetal brain data (Tanaka et al., 2020), yet each protocol exhibited a unique preference for early developmental bypasses. Moreover, organoids showed upregulated focal adhesion/ER stress genes and downregulated aerobic respiration relative to the fetal brain, reflecting the impact of limited vascularization. In a parallel meta-analysis of later-time-point organoids (Kiaee et al., 2021), the 3-month organoids largely recapitulated fetal brain functional gene sets, but by 6 months they displayed significant deficits in axon guidance, synaptic transmission, and action potential enrichment compared to age-matched fetal tissue, suggesting a delayed or impaired functional maturation. These findings caution against the uncritical use of long-term organoids for modeling late-stage neural circuitry and highlight the need for protocol refinements to achieve more faithful functional development.

Notwithstanding current progress, challenges persist in modeling late-stage tauopathy and sAD. Organoids lack 4R tau isoforms and show immature gene expression patterns that differ from adult neurons. Therefore, excessive mutant tau expression must be used to create aggregation events (Schrank et al., 2020; Batenburg et al., 2023). The combination of microfluidics with multi-omics methods will solve existing knowledge gaps because this will enable the monitoring of tau-induced gene expression changes and single-cell responses at different time points (Anand et al., 2022; Batenburg et al., 2023). Tri-culture systems must continue to be created and include microglia cells to study their involvement in neuroinflammation and their ability to transfer tau proteins. Research must also concentrate on developing vascularized organoids through bioengineering to study how BBB challenges affect tau protein spread (Hofer and Lutolf, 2021; Batenburg et al., 2023).

Assembloids are used in combination with 3D co-culture systems for a new scientific approach that connects laboratory-based studies with human disease mechanisms. The platforms enable investigation into disease mechanisms and therapeutic targets by enabling model tau protein spread between brain circuits and its interactions with specific cell types. The tools show high scalability and full compatibility with patient-derived cells, making them vital for personalized medicine and drug discovery applications in neurodegenerative diseases (Hofer and Lutolf, 2021; Anand et al., 2022; Batenburg et al., 2023).

### 4.3 Neuroinflammation and glial cell dysfunction

Organoid models have enabled scientists to study microglia functions in synaptic elimination and Aβ plaque removal, advancing our understanding of AD development through models that demonstrate neuroinflammatory reactions and glial cell destruction. Organoids, which stem from human iPSCs, connect traditional 2D cell cultures to real-life models through their 3D structure that supports natural cell-to-cell interactions between neurons, astrocytes, and microglia (Guido et al., 2023; Fixemer et al., 2025; Wendt et al., 2025). These systems show how microglia cells respond to Aβ through different stages, leading to neurotoxic behavior from their initial neuroprotective state (Kang et al., 2024; Holmes et al., 2025; Wendt et al., 2025). The microglial cells in organoid cultures phagocytose Aβ oligomers, resulting in decreased plaque formation and decreased oxidative stress. The normal process of synaptic pruning becomes disrupted because of ongoing inflammation, which results in the excessive destruction of synapses and damage to neurons (Fixemer et al., 2025; Holmes et al., 2025). The two distinct microglial populations in the brains of AD patients show different characteristics through their identification as disease-associated microglia (DAM). These cells include plaque-associated (PaM) and coffin-like (CoM) microglia, presenting distinct molecular characteristics and specific locations in hippocampal regions (Huch, 2023; Fixemer et al., 2025). The PaM region, which contains Aβ plaques, shows increased gene expression of complementary pathways and lipid metabolism genes; however, CoM exists in pyramidal layers where it links to tau-related neuronal destruction (Fixemer et al., 2025). TREM2 and APOE receptors located on microglial cells determine their ability to perform phagocytosis, and APOE4 variants in APOE result in decreased Aβ clearance and elevated brain inflammation (Mancuso et al., 2024; Fixemer et al., 2025).

The combination of electrophysiological recordings with single-cell RNA sequencing data reveals that organoids that receive microglia through two different methods develop better neuronal development and synaptic function and network stability (Fagerlund et al., 2021; Wendt et al., 2025). Intestinal stem cell development becomes more advanced through NRG1 produced by microglia, suggesting that AD neuronal protection follows a similar mechanism (Yu et al., 2021). Organoids enable the study of microglia–astrocyte interactions through high-content imaging and spatial transcriptomics, revealing that APOE from astrocytes functions as a key factor in microglial deterioration in AD (Fixemer et al., 2025; Wendt et al., 2025). CRISPR-engineered organoids are utilized to study AD risk genes, showing their effect on microglial activation patterns and their ability to remove Aβ (Mancuso et al., 2024; Fixemer et al., 2025).

However, multiple barriers exist that block success. Organoid models require essential development for two main reasons: prevention of hypoxia-induced necrotic damage and the requirement for vascularization to achieve better physiological characteristics (Fagerlund et al., 2021; Hofer and Lutolf, 2021). Future research should include two main directions: neuroimmune interactions through organoid-based immune component integration and BBB modeling using microfluidic systems (Yuki et al., 2020; Hofer and Lutolf, 2021). Organoid models can also be used for customized drug testing and new treatments for tissue repair. These models will totally revolutionize the scientific investigation of neurodegenerative diseases.

### 4.4 BBB disruption and vascular pathology

Organoid models function as a research platform for AD studies because they reproduce the fundamental pathological elements of the disease, including vascular damage and BBB disruption. The blood–brain barrier (BBB) is a physical and biochemical barrier composed of endothelial cells linked by tight junctions, with pericytes embedded in the basement membrane and astrocytic endfeet providing functional support (González-Hernández et al., 2026); it regulates the passage of molecules between the blood and the brain parenchyma. In contrast, the neurovascular unit (NVU) is a more comprehensive concept that encompasses the BBB plus adjacent neurons (including interneurons), microglia, and the basement membrane, emphasizing the dynamic coupling between neuronal activity and local blood flow (neurovascular coupling) as well as metabolic support. Thus, BBB dysfunction is a key event within the broader context of NVU pathology (He and Sun, 2025). Cerebral amyloid angiopathy (CAA) can be researched through vascularized organoid systems because this condition develops when amyloid-beta (Aβ) accumulates in blood vessel walls of the brain, leading to cerebrovascular problems (Durrant et al., 2020; Deng et al., 2022; Steiner and Humpel, 2023). These models preserve the neurovascular unit (NVU) cytoarchitecture, which contains ECs, pericytes, astrocytes, and neurons to maintain BBB structure and allows Aβ removal, thus facilitating laboratory tests and animal studies (Kamikubo et al., 2023; Xiong et al., 2025). Aβ40 and Aβ42 peptides block endothelial cell movement while causing abnormal blood vessel development in brain tissue cultures derived from transgenic mice, displaying the first vascular changes that occur in AD and CAA (Deng et al., 2022; Steiner and Humpel, 2023). The combination of human iPSC-derived neurons and ECs in microfluidic-based vascularized organoids shows that Aβ causes tight junction disruption, resulting in BBB permeability. This research identifies vital information about blood vessel Aβ accumulation leading to increased brain cell deterioration (Uzoechi et al., 2024; Xiong et al., 2025).

The ability of these models to replicate the temporal and spatial dynamics of Aβ deposition in vessel walls is a significant innovation. Bioengineered cerebral vessels that include astrocytes and neurons show Aβ40 fibril accumulation in their vascular walls at elevated levels compared to other regions, which corresponds to the Aβ40:Aβ42 ratio discrepancy observed in CAA patient tissue samples (Chiu et al., 2022; Xiong et al., 2025). The 3D microvascular networks on organ-on-chip platforms show that Aβ oligomer-induced endothelial cell death and barrier disruption occurs at different doses (Robert et al., 2020; Uzoechi et al., 2024). The systems demonstrate how RAGE-mediated Aβ trafficking and LRP1 dysregulation create conditions that continue to advance vascular disease (Chiu et al., 2022; Xiong et al., 2025). Furthermore, research on vascularized organoids has demonstrated Aβ-activated neuroinflammatory responses through experiments, demonstrating that astrocytes with increased GFAP and AQP4 protein expression develop vascular amyloidosis corresponding to human CAA disease severity (Daoutsali et al., 2023; Xiong et al., 2025).

Organoid vascularization has received additional attention through recent technological advances. Self-organizing microvascular networks can be created by combining fibrin-Matrigel hydrogels with aprotinin and bFGF, which function independently from fibroblast cells for support (Wen et al., 2024). The hydrogels contain engineered collagen fiber bundles that guide vascular network formation to create brain microenvironment-like structures to improve organoid blood flow (Wen et al., 2024). The development of perfusion-based microfluidic technologies allows the study of Aβ trafficking and barrier permeability at fast speeds while minimizing experimental mistakes. The new technologies operate alongside current scientific advances (Uzoechi et al., 2024; Xiong et al., 2025). CAA-related genetic factors can be studied through vascularized organoids that develop Aβ42 deposits and tau phosphorylation changes at an accelerated rate in patients with AD with the APOE ε4 genotype (Kim et al., 2024; Xiong et al., 2025).

These models enable researchers to evaluate different treatment approaches because they possess translational value. The inhibition of BACE1 in TgCRND8-derived OBSCs results in NOTCH3 signaling restoration and normal angiogenesis patterns, which indicates that BACE1 could function as a therapeutic agent for CAA treatment (Durrant et al., 2020; Xiong et al., 2025). The testing of anti-Aβ immunotherapies in vascularized organoids enables the determination of the effectiveness of decreasing vascular amyloid deposits and fixing damage to the BBB (Santos et al., 2024; Xiong et al., 2025). Microglia cells in cerebral organoids (COiMg) function as brain inflammation regulators during Aβ exposure. IFN-γ exposure leads to changes in genes associated with autism, and studies have elucidated the movement of microglial cells (Buonfiglioli et al., 2025; Sarnow et al., 2025).

Vascularized organoids serve as two functional tools that enable the investigation of biological systems and the assessment of therapeutic candidates. A microfluidic, membrane-free BBB model using human endothelial cells showed that Aβ1-42 oligomers induce tight junction disruption (ZO-1), increased barrier permeability (FITC-dextran), and endothelial cell death in a dose-dependent manner (LC50 ∼1 μM). This model captures key aspects of Aβ-induced BBB dysfunction, including vascular Aβ deposition, but does not address APOE genotype or tau pathology (Uzoechi et al., 2024). Directly reprogrammed 3D brain organoids derived from human fibroblasts express neuronal markers and form synapses within 20 days. Organoids from APOE ε4 AD patients exhibit increased Aβ42 deposition, elevated phosphorylated tau (p-Tau), and thioflavin T-positive aggregates compared to APOE ε3 controls. The co-expression of mutant APP further accelerates these pathologies. Transcriptomic analysis reveals overlaps with APOE ε4 post-mortem AD brain signatures. However, this model lacks vascular components and does not study BBB function (Kim et al., 2024). Nevertheless, as discussed in detail in Section 7.1, these models have important limitations—most notably, they lack functional perfusable vessels with active blood flow, which restricts their ability to model chronic hypoperfusion, glymphatic clearance, and age-dependent vascular remodeling. Therefore, while vascularized organoids represent a significant advance over non-vascularized systems, they should be considered complementary tools rather than a complete “new research standard” for all aspects of AD cerebrovascular pathology. The tools require patient cells, microenvironmental changes, and genetic risk factors to detect disease origins and accelerate treatment development. Research should further investigate how different organoid systems affect cerebrovascular diseases in multiple body organs and develop methods to create blood vessels within organoids.

## 5 Unveiling new perspectives on AD pathogenesis

### 5.1 Genetic mutations and disease mechanisms

Research has made significant progress in identifying genetic defects that cause AD through the study of amyloid precursor protein (*APP*), *PSEN1*, and *PSEN2* genes. These genes serve essential roles that lead to inherited AD and early-onset AD (EOAD) and affect disease progression and medical intervention.

The amyloid precursor protein (APP) gene exists on chromosome 21. Mutations to this gene produce distinct functional abnormalities in cerebral organoids through modifications of APP processing, resulting in AD pathological features. The Swedish (K670N/M671L), London (V717I), and Arctic (E693G) mutations are concentrated in exons 16 and 17 (the Aβ-coding region and surrounding areas). Organoids exhibit stable expression of mutant APP. The amyloidogenic processing pathway of APP becomes more active because specific mutations create stronger binding sites that enable APP to bind more strongly with β-secretase (BACE1) (Chen Jiang et al., 2023). The APP mutations lead to an elevated ratio of Aβ42 to Aβ40. The Aβ42 peptide shows better aggregation behavior, which results in amyloid plaque formation that exactly resembles the brain plaques found in patients with AD (Gonzalez et al., 2018; Chen Jiang et al., 2023).

Cerebral organoids function as a biological system that enables researchers into APP gene mutations to create AD and amyloidopathy models to understand disease processes and develop new treatments. The main features of AD are studied through 3D human brain microenvironments developed using induced pluripotent stem cell (iPSC)-derived organoids (the Dominantly Inherited Alzheimer Network DIAN et al., 2018; Daoutsali et al., 2023; Kim et al., 2024). Cerebral organoids that stem from Dutch-type cerebral amyloid angiopathy (D-CAA) patients who have the APP E693Q mutation show rapid Aβ40 accumulation starting at 52 days of culture development. The Aβ40 aggregation process in these models occurs faster than in conventional AD models that require multiple months of development (Daoutsali et al., 2023). The Aβ domain contains this mutation, which changes peptide hydrophobic properties to create fibrils that accumulate in blood vessels. This phenotype is accurately replicated in D-CAA organoids without the need for exogenous Aβ overexpression (Daoutsali et al., 2023). The organoids contain elevated levels of neuronal and astrocytic markers together with disrupted TGF-β signaling, suggesting that APP mutations cause neuroglial homeostasis disruption and amyloidogenesis (Daoutsali et al., 2023).

Organoids are useful for the study of both protective and sporadic APP variations in addition to familial AD. Current research investigates how the APP A673T mutation, which reduces Aβ production and accumulation, protects against AD (Xia et al., 2021). The Shanghai APP mutation in a late-onset AD patient resulted in better β-secretase (BACE1) cleavage efficiency, which produced higher amounts of Aβ42 in laboratory cell cultures (Zhang et al., 2024). The AAV-mediated expression system in mice produced results that confirmed both cognitive deficits and amyloid deposition in mice through the E674Q mutation, disrupting APP-BACE1 protein interface stability according to molecular dynamics simulations (Zhang et al., 2024). Organoids have proven valuable for studying how genetic variations affect disease symptoms according to research findings that focus on mutations near the β-secretase cleavage site; here, mutations create structural changes that strongly affect how the body produces amyloidogenic proteins (Xia et al., 2021; Zhang et al., 2024).

Organoids developed through patient fibroblast direct reprogramming maintain their epigenetic marks, enabling them to keep age-related characteristics that make them better for disease studies (Kim et al., 2024). APOE ε4-induced organoids have been utilized to study how mutant APP introduction creates AD-related gene expression patterns, matching results in AD brain tissue after death (Kim et al., 2024). The London mutation in APP (APP V717I) causes decreased neurite growth and enhanced synaptic problems; it has been studied through CRISPR/Cas9 genome editing of isogenic organoids that express LINGO2 and other genes (Qu et al., 2024). A single-nucleus RNA sequencing analysis of these grafts showed which cells suffered the most damage through its examination of APP functions in synaptic maintenance and axonal guidance (Qu et al., 2024).

Organoids have also been creatively used to investigate non-amyloidogenic APP functions. The PI3K/Akt pathways became active when APP levels exceeded normal limits in neuronal models that protect mitochondria from damage. The reduction of APP expression in neural stem cells triggered changes in β-catenin signaling, leading to neurogenesis becoming more prominent than gliogenesis during cell differentiation (Cimdins et al., 2019; Coronel et al., 2023). APP mutations create multiple effects that reach further than amyloid-β (Aβ) generation because they appear to affect brain development, cell communication, and the body’s response to oxidative stress (Cimdins et al., 2019; Coronel et al., 2023).

Organoids stemming from human iPSCs offer researchers the best possible understanding of disease-specific features because they duplicate the human brain’s 3D organization and multiple cell types. fAD iPSCs with *PSEN1* or *PSEN2* mutations used to generate retinal organoids develop AD-related features, including Tau protein hyperphosphorylation (p-Tau) and elevated Aβ42:Aβ40 ratios that match initial retinal changes in patients (Lavekar et al., 2023). The models show that the retina contains multiple biomarkers that can be used to identify AD at its beginning stages and to confirm that presenilin gene mutations result in amyloid protein production (Lavekar et al., 2023). The P436S mutation in PSEN1 leads to abnormal γ-secretase enzyme function, resulting in cortical organoids producing elevated levels of Aβ43. This finding is consistent with observations from post mortem analyses of patient brains (Arber et al., 2024). The *PSEN1* P436S mutation, which occurs in the essential PALP motif of the protein, causes substrate handling problems but allows normal protein development, revealing the specific structural elements that cause presenilin protein damage (Arber et al., 2024).

The study confirmed pathogenicity through its analysis of *PSEN1* V142F and G206D mutations, which affect Aβ42:Aβ40 ratios and Pen-2 expression (Pimenova and Goate, 2020). The use of iPSC-derived neuronal models showed that *PSEN1* L435F heterozygosity results in higher Aβ43 concentrations and increased p-Tau levels, which demonstrate that presenilin dysfunction causes tauopathy without affecting traditional Aβ40/42 ratios (Oakley et al., 2020). These findings challenge the amyloid cascade hypothesis because they suggest that γ-secretase functional decline instead of amyloid accumulation leads to neurodegenerative processes (Oakley et al., 2020). The PSEN1 mutation, which removes phospho sites from microglia cells, leads to their inability to properly break down Aβ oligomers in phagolysosomes, resulting in increased synaptic damage in AD mouse models (Ledo et al., 2021). Furthermore, presenilins operate as non-cell-autonomous elements that control neuroinflammation and clearance processes.

PSEN1 mutations located beyond codon 200 are associated with lower amyloid burden but more severe small vessel disease (SVD) and earlier cognitive decline than mutations occurring before codon 200. These differences have been demonstrated in Columbian kindreds using neuroimaging modalities, including diffusion tensor imaging (DTI) (Joseph-Mathurin et al., 2024). The genetic classification system enables the forecasting of treatment results by creating new clinical tests to evaluate anti-amyloid medications. Organoids serve as tools for conducting large-scale tests of γ-secretase modulators (GSMs), enabling studies of *PSEN1*-containing γ-secretase bound to MRK-560 through cryo-EM. These models may identify Thr281 and Leu282 as key factors for developing specific and secure therapeutic agents (Guo et al., 2022). Disease progression of retinal organoids can also be tracked because these studies do not require invasive procedures to perform transcriptional profiling, revealing that synaptic dysfunction pathways activate before other AD-related changes are visible (Lavekar et al., 2023).

Mutations in the four EOAD genes result in identical biological mechanisms that affect APP processing. These mutations create small changes in protein function that cause problems with APP processing, leading to AD development (Rademakers et al., 2003). Transgenic mouse models have been utilized to study EOAD mutations inside living organisms, helping us understand how these mutations affect disease processes and develop new treatment methods. Multiple new mutations occur in these genes. Gan et al. (2020) identified mutation sites in APP, PSEN1, and PSEN2 genes that occur in Chinese people and could affect AD. Furthermore, Takada et al. (2022) discovered multiple PSEN1 mutations in Latin American populations and established the genetic changes most likely to cause disease progression in AD patients. Furthermore, genetic diversity between populations plays a crucial role in understanding the genetic factors that contribute to Alzheimer’s disease.

The PSEN1 M139L mutation, which resides in the transmembrane α-helix of the protein, was studied to determine its impact on Aβ42/Aβ40 ratio elevation leading to amyloid plaque formation (Qiu et al., 2019). The variability in clinical presentation associated with different mutations has also been a focus of recent research. Fernández et al. (2025) noted that age at symptom onset (AAO) remains relatively consistent among PSEN1 mutation carriers but varies more in PSEN2 and APP variants, which tend to be associated with later AAOs. The extent of γ-secretase dysfunction thus determines the delay in disease progression by PSEN1 mutations that cause complete enzyme inactivation.

Somatic mutations and genetic mosaicism in AD have been suggested by new data from genomic analyses. Li et al. (2025) found that neurons contained more somatic mutations that included AD-related genes APP, PSEN1, and PSEN2, indicating that these mutations could play a role in neurodegenerative processes and disease advancement. Research should analyze both inherited and somatic genetic changes to understand the complete biological processes leading to AD. The operational mechanisms of these mutations require further research to analyze the various symptoms that appear in patients and to evaluate their potential as therapeutic agents. AD genetic analyses require advances through the combination of functional studies with genetic population research and mutation analysis in human tissues.

#### 5.1.1 Application of gene editing techniques

Brain organoids have undergone a transformation in the study of neurodevelopment and disease modeling and therapeutic screening through the use of CRISPR/Cas9 gene editing tools that introduce specific gene mutations. Brain organoids derived from induced pluripotent stem cells (iPSCs) or embryonic stem cells (ESCs) function as essential laboratory instruments used to investigate genetic mutations because these structures mimic brain tissue and contain brain-like cellular components that replicate human brain organization (Fischer et al., 2019). The CRISPR/Cas9 tool is used in the study of neurodevelopmental disorders, neurodegenerative diseases, and evolutionary changes because it enables precise and efficient targeted modifications, including small single-nucleotide alterations and large gene insertion or deletion operations (Fischer et al., 2019; Gallego Villarejo et al., 2024). CRISPR/Cas9 is used to knock out disease-related genes in cerebral organoids to study glioblastoma stem-like cells (GSCs) and neural stem/progenitor cells. The method produces more than 90% indel mutations that occur within a short period of time for loss-of-function studies without needing to establish clonal cell lines (Hoellerbauer et al., 2020). The fast and deep editing function serves as an essential requirement for conducting large-scale functional genomics studies and drug development programs.

The main advantage of using CRISPR/Cas9 in brain organoids stems from its ability to work with multiple delivery methods from which researchers can choose based on their specific experimental needs. The study of gene function requires persistent modifications achieved through lentiviral vectors, transposon systems, and homology-directed repair (HDR) for extended research periods. The research of short-term gene expression requires the use of AAV and electroporation as a preferred transient method (Fischer et al., 2019; Gallego Villarejo et al., 2024). The method of electroporation has been optimized to work with primate cerebral organoids, allowing the targeting of ventricle-like structures for ectopic gene expression while keeping toxicity levels low; however, the optimal plasmid concentration needs to be obtained to prevent cell death (Tynianskaia et al., 2023). The delivery of CRISPR/Cas9 ribonucleoprotein (RNP) complexes through nanoblades (NBs) produces superior results (75% knockout success in murine organoids) while preventing unwanted gene modifications, which traditional electroporation methods fail to achieve for sustained genetic changes (Tiroille et al., 2023). The method uses virus-like particles to show Cas9/sgRNA expression, resulting in minimal DNA changes and enabling the study of phenotypes over a short period (Tiroille et al., 2023).

The CRISPR/Cas9 system can be utilized to perform knockout studies, edit DNA bases, and control gene expression in brain organoids. Furthermore, single-cell electroporation can be used to deliver CRISPR/Cas9 and base editors (BEs) into Purkinje cells, which enables the detailed study of circuits through targeted gene disruption and nucleotide conversion (Song et al., 2021). The CRISPR activation (CRISPRa) system for post-mitotic neurons operates as an endogenous gene expression mechanism, allowing specific Bdnf transcript variants to be boosted while leaving other genes unaltered in the study of particular isoforms in synaptic plasticity (Savell et al., 2019). Traditional overexpression vectors face two major restrictions: they have size limitations and they cannot fit inside viral packaging systems, which prevents them from performing precise genetic modifications (Savell et al., 2019).

The modified organoid models achieve dependability through the combination of CRISPR/Cas9 technology with optical genome mapping and other advanced genomic quality control systems. CRISPR-edited iPSCs maintain their pluripotent properties and organoid development capabilities; however, validation is required due to the editing procedure generating genomic abnormalities, including copy number variations (Gallego Villarejo et al., 2024). The simulation of diseases requires special attention because small genetic changes may produce effects that exacerbate disease symptoms. The IL-17 signaling genes NFKBIZ and ZC3H12A develop somatic mutations that occur in ulcerative colitis organoids. These cells demonstrate the inflammatory conditions that affect the process of clonal selection, revealing disease mechanisms (Nanki et al., 2020).

The flexible gene editing system of CRISPR/Cas9 and its related technologies enable researchers to study gene function, disease mechanisms, and therapeutic targets through precise mutation induction in brain organoids. The benefits of these tools include single-cell analysis, high efficiency, and scalability, which have transformed neurological studies. However, to fully realize their potential, meticulous delivery technique optimization and rigorous genomic validation are still necessary. Research should progress through the combination of CRISPR technology with optogenetics and assembloid systems to create models that show how different brain regions interact with each other (Tynianskaia et al., 2023; Hong S. et al., 2024).

### 5.2 Cell interactions and disease progression

#### 5.2.1 Neuron and glial cell signaling

The rapid advance of multi-omics technology shows how cerebral organoids preserve intricate neural-glial relationships that control brain development in the study of neurological diseases. Organoid research has been advanced through proteomic methods, revealing protein networks that direct synaptic development, axon pathfinding, and glial cell formation. Mass spectrometry-based processes enable the detection of brain tissue markers and lipid profiles, assisting in the creation of detailed maps of cell-type-specific connections (Nascimento et al., 2019; Nezvedová et al., 2023). A proteomic study of 45-day-old organoids identified two vital pathways in which glypican signaling and SPARCL1/hevin-mediated synaptogenesis were used to facilitate neuron–glia communication (Nascimento et al., 2019). The organoids consist of calcium-binding proteins and NMDA-receptor-associated proteins, which validate the crucial function of calcium signaling and glutamate/glutamine cycles in astrocyte–neuron metabolic coupling (Nascimento et al., 2019). The data support the transcriptomic results, showing that Notch and BMP pathways become more active in astrocytes while regulating neuronal development and synaptic elimination processes (Kiaee et al., 2021; Werner and Gillis, 2024).

Single-cell RNA sequencing (scRNA-seq) enables the investigation of neuron–glia signaling patterns through different developmental stages. CLU and METRN proteins exist only in astrocytes because these cells need them to develop neurites and maintain their network structure. Organoid cells function as excitatory neurons, and astrocytes express their axon guidance genes in a synchronized manner (Kiaee et al., 2021; Werner and Gillis, 2024). Patch-clamp and multielectrode array (MEA) recording results have shown that the development of microglia-like cells from erythromyeloid progenitors (EMPs) in organoids leads to better neuronal development because these cells support both neuronal firing repetition and gamma-band oscillation generation (Fagerlund et al., 2021). The functional integration between microglia and neurons becomes evident through proteomic data, demonstrating that microglia perform synaptic pruning through complement cascade proteins (Nascimento et al., 2019; Fagerlund et al., 2021).

New regulatory systems have been discovered by using multi-omics methods. Research on gliogenesis using lipidomics has revealed that cells change their surface lipids; however, proteomic and transcriptomic analyses have demonstrated that L1CAM and ROBO1 function as essential proteins for neuronal migration under radial glia direction (Nascimento et al., 2019; Nezvedová et al., 2023). Furthermore, spatial omics methods have been used to identify the separate locations of signaling niches, including Notch-activated astrocytes in subventricular zones and Wnt/BMP-responsive progenitors in ventricular zones (Nascimento et al., 2019; Werner and Gillis, 2024). Electrophysiological tests have supported these findings, showing that organoids with microglia-like cells developed sophisticated synaptic functions and long-term potentiation (LTP) patterns that mimic natural neuronal networks (Zafeiriou et al., 2020; Fagerlund et al., 2021).

Current research does not, however, solve modern problems that impact organoid manufacturing dependability and blood vessel development in organoid culture systems. Deep organoid layers require blood vessel development to receive nutrients for proper signal transmission (Kiaee et al., 2021; Werner and Gillis, 2024). However, bioprinting enables investigation into neuron–glia interactions for extended periods through the development of vascularized organoids. CRISPR-based disruption screens need to be combined with multi-omics analysis to discover the vital mechanisms that regulate these processes (Picard and Shirihai, 2022; Pan et al., 2024). Multi-omics technologies achieve two main goals: reading the molecular language of neuron–glia signaling in organoids and creating exact models of neurodevelopmental disorders and testing new treatments.

#### 5.2.2 Neurovascular unit (NVU) dysfunction

While Section 4.4 addressed BBB breakdown as a pathological endpoint, this subsection focuses on the integrated dysfunction of the NVU—that is, the coordinated failure of neurons, glia, and vessels that underlies neurovascular uncoupling, metabolic deficits, and impaired Aβ clearance in AD. The NVU in cerebral organoids serves as a vital research domain for creating enhanced laboratory models to duplicate brain development and disease processes through the development of functional structures, including blood vessel formation, BBB function, and neuronal growth factors. VOs produce better BBB-like properties because their endothelial cells organize into tubular patterns that display CD31 expression and permeability properties to match the natural BBB of living organisms (Pham et al., 2018; Faley et al., 2019). Three-dimensional models use iPSC-derived brain microvascular endothelial cells under perfusion conditions to maintain their barrier function for extended periods because tight junction proteins claudin-5 and occludin remain correctly positioned. Furthermore, endothelial gene expression levels that are lower than those in natural tissue standards require additional improvement (Faley et al., 2019; Fumadó Navarro et al., 2025).

Organoids develop new blood vessels through hypoxia-activated signaling pathways such as HIF-1 and angiopoietin-2 proteins that attract endothelial cells from perineural vascular plexuses to create new blood vessels during development (Pham et al., 2018; Shi et al., 2020). The use of HUVECs or iPSC-derived ECs in co-culture systems results in brain-like EC phenotypes that express P-glycoprotein and form vascular networks that link organoid areas to reduce core area hypoxia and apoptosis (Shi et al., 2020; Fumadó Navarro et al., 2025).

Organoids receive enhanced neuronal trophic support because ECs release VEGF, angiopoietin-1, and BDNF, which collectively stimulate neurogenesis and synaptic development (Shi et al., 2020; Cho et al., 2021). The VOs demonstrate the accelerated development of mature neuronal markers and synaptogenesis compared to non-vascularized controls (Cho et al., 2021; Fumadó Navarro et al., 2025). The trophic relationship between cells becomes more evident through the combination of increased organoid dimensions and decreased caspase-3-positive cell numbers, showing how vascular networks support metabolic requirements (Shi et al., 2020; Cho et al., 2021). The establishment of functional blood flow and vascular connections between organoids remains difficult to achieve in laboratory studies; however, host blood vessels can link with transplanted organoids to create vascular connections, suggesting further investigation in future medical uses (Pham et al., 2018; Wörsdörfer et al., 2019).

The development of organoid architecture has progressed through microfluidic system advances that incorporate brain-specific extracellular matrices to improve oxygen/nutrient supply, minimize necrotic core development for enhanced NVU representation and extended culture periods, and complete functional development (Cho et al., 2021; Fumadó Navarro et al., 2025). The systems show particular interactions with laminar tissue because their Müller glia cells surrounding capillaries in retinal organoids behave like BBB astrocytic endfeet and exhibit purinergic stimulus-induced Ca^2+^ signaling activity (Grimes et al., 2025). However, barrier strength is reduced because pericytes fail to provide adequate protection, and the pattern of blood vessel distribution remains unclear (Faley et al., 2019; Shi et al., 2020).

Circadian rhythms also affect metabolic processes in NVU models. The optimal NVU component mix between pericytes and astrocytes still requires elucidation to produce stable angiogenic sprouts and improve gene expression results (Faley et al., 2019; Li W. et al., 2024). The research into neurovascular interactions using vascularized organoids has included a groundbreaking platform that allows neurodegenerative diseases and test treatments to be studied; however, the framework requires further development to fully replicate *in vivo* conditions.

### 5.3 Key signaling pathway analysis

The Wnt/β-catenin and PI3K/Akt signaling pathways serve as critical elements for AD progression, and these pathways develop abnormalities in organoid models that exhibit AD characteristics. Organoids made from iPSCs of AD patients who have *PSEN1* E280A mutations show Wnt/β-catenin signaling problems because their β-catenin levels decrease and GSK3β activity increases. This results in tau protein hyperphosphorylation and neurofibrillary tangle development (Perugorria et al., 2019; Perez-Corredor et al., 2024). Furthermore, APOE3 Christchurch (APOE3Ch) genetic variation enhances Wnt/β-catenin signaling in cerebral organoids, which results in reduced tau phosphorylation and decreased *PSEN1* mutation-induced disease progression. Therefore, Wnt activation provides protection against AD (Perez-Corredor et al., 2024; Song et al., 2024). A different version of the protein decreased tau hyperphosphorylation while maintaining β-catenin stability through its effect on protein translation, resulting in modifications of cadherin and Wnt signaling pathways (Perez-Corredor et al., 2024). The Wnt/β-catenin pathway showed elevated activity in the frontal cortex of APOE3Ch *PSEN1* E280A carriers, demonstrating a protective effect that depended on the amount of APOE3Ch present (Perez-Corredor et al., 2024). These results highlight the potential of Wnt/β-catenin regulation as a therapeutic approach to prevent the progression of AD.

The PI3K/Akt pathway, which controls cell survival and synaptic plasticity, becomes disrupted when AD causes the failure of neurotrophic support and rise in neuronal death. The natural coumarin osthol shows neuroprotective properties for AD animals by changing the PI3K/Akt signaling pathway, leading to decreased Aβ-mediated synaptic protein breakdown and enhanced mental performance (Ji et al., 2021; Hu et al., 2023). The two signaling pathways use glycogen synthase kinase-3β (GSK3β) as a shared target to stabilize neurons through their combined Wnt and PI3K/Akt signaling pathways. The development of new therapeutic methods shows promise of achieving two goals: restoring route equilibrium and decreasing AD pathology through GSK3β small-molecule inhibitors and Wnt activators such as APOE3Ch mimetics (Liang et al., 2022; Perez-Corredor et al., 2024). Organoid models function as a research instrument that allows investigation into these mechanisms while creating therapeutic approaches that can transition from laboratory discoveries to medical applications.

The multiple signaling pathways between Aβ and Wnt and inflammatory and oxidative stress systems create a complex pathophysiology that characterizes AD. Aβ oligomers cause synaptic damage, which results in cognitive impairment because they interact with receptors including PrPC, NgR1, and LilrB2 (Smith et al., 2019). The Wnt signaling pathway becomes dysregulated because Aβ triggers Dkk1 expression, which shifts the system from its normal Wnt/β-catenin (synapse-stabilizing) to its non-canonical Wnt-PCP (synapse-destabilizing) pathway, leading to an increased destabilization of synapses (Elliott et al., 2018). The APP protein serves as a co-receptor that participates in both signaling pathways. The Swedish mutant version of APP, known as “APPSwe”, causes synaptic loss to occur faster because it reduces the normal Wnt signaling pathway while enhancing Wnt-PCP pathway activity (Elliott et al., 2018). The inflammatory response becomes more severe because Aβ activates microglia and astrocytes to generate pro-inflammatory cytokines, activating the p38 MAPK and NF-κB pathways to increase neurodegenerative damage (Li et al., 2022; Mishra and Kumar, 2025). The cytokines generate reactive oxygen species (ROS), which extend the duration of oxidative stress while simultaneously increasing tau hyperphosphorylation through GSK3β activation (Maurice and Angers, 2025; Mishra and Kumar, 2025).

The initial stage of AD development brings about oxidative damage, which disrupts DNA repair mechanisms and calcium regulation through CAMK1 activity. Furthermore, Aβ generates ROS that create a self-reinforcing process of neuronal tissue breakdown (Choi et al., 2025; Maurice and Angers, 2025). The IGF1R/INSR hybrids operate through Aβ protein interaction with β-arrestin1, which activates ERK signaling and breaks down cAMP control systems to harm neuronal survival pathways. The process shows how different signaling pathways merge into a single operational system (Sengupta and Mukhopadhyay, 2025).

#### 5.3.1 Microglial-mediated synaptic pruning and synapse loss pathways

AD is characterized by early and progressive synapse loss that correlates more strongly with cognitive decline than amyloid plaque burden. Emerging evidence implicates the classical complement cascade as a key mediator of CNS synapse elimination during development. Stevens et al. (2007) demonstrated that C1q, the initiating protein of the classical complement cascade, is expressed by postnatal neurons in response to signals from immature astrocytes and is localized to synapses throughout the developing CNS and retina. Mice deficient in C1q or the downstream complement protein C3 exhibit sustained defects in retinogeniculate synapse elimination, as shown by the failure of eye-specific segregation and the persistence of multiply innervated lateral geniculate neurons. These findings support a model in which unwanted synapses are tagged by complement proteins (C1q and C3) for elimination. Although the precise cellular mechanism remains to be fully elucidated, the C3b-opsonized synapses are likely phagocytosed by resident microglia, the primary phagocytic cells in the brain that express C3 receptors (CR3). This complement-dependent mechanism operates during a discrete developmental window that coincides with the presence of immature astrocytes and active synaptic pruning (Stevens et al., 2007). Using a combination of gene profiling, immunohistochemistry, and electrophysiology in the mouse retinogeniculate system, Stevens et al. (2007) identified an unexpected role for astrocytes and the classical complement cascade in mediating CNS synapse elimination. They demonstrated that C1q, the initiating protein of the classical complement cascade, is expressed by postnatal neurons in response to signals from immature astrocytes and is localized to synapses throughout the developing CNS and retina during a discrete developmental window (P4–P10) that coincides with active synaptic pruning. Mice deficient in C1q exhibited sustained defects in eye-specific segregation and synapse elimination, as shown by the anatomical refinement failure of retinogeniculate connections and the retention of excess retinal innervation by lateral geniculate neurons. Specifically, C1q KO mice at P30 showed 81% of LGN neurons that remained multiply innervated (with four or more inputs), compared to most wild-type neurons receiving only one or two inputs. These deficits were not attributable to changes in retinal ganglion cell number or early patterned activity. Crucially, mice deficient in the downstream complement protein C3 displayed remarkably similar phenotypes, with 77% of LGN neurons classified as “resolving” (multiply innervated) at P30, demonstrating that the classical complement cascade as a whole, rather than C1q alone, mediates developmental synapse elimination. Therefore, unwanted synapses may be tagged by complement proteins (C1q and C3) for elimination, and C3b-opsonized synapses are likely phagocytosed by resident microglia, the primary phagocytic cells in the brain that express C3 receptors (CR3). Notably, complement-dependent synapse elimination represents only a portion of the overall pruning process, as complement-deficient mice still underwent some degree of synaptic refinement, indicating the existence of complement-independent mechanisms. These findings established the complement cascade as a critical mediator of developmental CNS synapse elimination and suggested that this mechanism may become aberrantly reactivated in neurodegenerative diseases such as AD and glaucoma, where C1q is profoundly upregulated (Stevens et al., 2007).

In AD, the developmental mechanism of complement-mediated synapse elimination is aberrantly reactivated, leading to the excessive elimination of healthy synapses. Hong et al. (2016) demonstrated that in the J20 mouse model of AD, C1q is upregulated as early as 1 month of age, prior to overt plaque deposition, and is localized to synapses in vulnerable brain regions including the hippocampus and frontal cortex. This synaptic C1q deposition precedes significant synapse loss, which becomes evident by 3–4 months of age. Using intracerebroventricular injections of soluble oligomeric Aβ (oAβ) into wild-type mice, oAβ but not monomeric Aβ induces rapid C1q deposition and co-localization with post-synaptic puncta (PSD95) within 72 h. Critically, oAβ failed to induce synapse loss in C1q-deficient mice, demonstrating that C1q is required for oAβ-induced synaptotoxicity. Furthermore, the co-administration of a function-blocking anti-C1q antibody (ANX-M1) prevented oAβ-induced synapse loss *in vivo* and rescued the impairment of long-term potentiation (LTP) in hippocampal slices, establishing C1q as a key mediator of synaptic dysfunction.

Furthermore, the classical complement cascade is activated downstream of C1q, as oAβ induces C3 deposition in wild-type mice, which was significantly reduced in C1q-deficient mice or by anti-C1q antibody treatment. In APP/PS1 mice, a higher percentage of synapses were colocalized with C3. Importantly, the genetic deletion of C3 in APP/PS1 mice (APP/PS1 × C3 KO) rescued synapse loss in the hippocampus, confirming that the full classical complement cascade mediates early synaptic degeneration. Mechanistically, microglia engulf synaptic material in response to oAβ challenge. Using Homer–GFP mice to label post-synaptic compartments, it was demonstrated that oAβ induces a significant increase in the volume of synaptic material internalized by microglia. This engulfment was dependent on the microglial complement receptor CR3, as oAβ failed to increase synaptic engulfment in CR3-deficient mice. Moreover, CR3 knockout mice were protected from oAβ-induced synapse loss, establishing that microglia eliminate synapses through a CR3-dependent mechanism. These findings established that complement-dependent microglial phagocytosis contributes directly to early synaptic degeneration in AD pathogenesis, occurring prior to plaque deposition and in response to soluble Aβ oligomers. Hong et al. (2016) provided the first direct evidence that the developmental synaptic pruning pathway is aberrantly reactivated in AD and identified complement proteins and microglia as potential early therapeutic targets.

Recent advances in brain organoid technology and xenotransplantation models have enabled the direct investigation of human microglial function in synaptic elimination and neurodegeneration. Using a combination of microglia-containing cerebral organoids and human-mouse microglial chimeras, Jin et al. (2022) demonstrated that Down syndrome (DS) microglia exhibit enhanced synaptic pruning function, as indicated by increased PSD95+ puncta within microglia in both organoids and chimeric mouse brains. This excessive synaptic elimination led to reduced dendritic spine density and impaired synaptic neurotransmission, including decreased mEPSC frequency and amplitude and impaired long-term potentiation (LTP) in DS microglial chimeras. Mechanistically, the overexpression of Hsa21-encoded type I interferon receptors (IFNAR1/IFNAR2) in DS microglia drives elevated IFN-I signaling, which underlies both enhanced developmental synaptic pruning and accelerated senescence in response to pathological tau. Single-cell RNA sequencing of DS microglia exposed to DSAD patient brain-derived pathological tau revealed a shift toward a senescent state rather than classical activation, with the upregulation of senescence-associated genes (*B2M*, *XAF1*, and *ZFP36L1*), downregulation of mitochondrial genes, and enrichment of interferon-stimulated genes (ISGs). Importantly, knockdown of IFNAR1/2 by shRNA rescued the DS microglial phenotypes, reducing synaptic pruning, restoring synaptic function, and preventing tau-induced senescence. Therefore, elevated IFN-I signaling drives both developmental synaptic over-pruning and pathological tau-induced senescence in DS microglia, providing a mechanistic link between trisomy 21, microglial dysfunction, and the accelerated progression of AD in DS (Jin et al., 2022).

Using high-content neuropathology-integrating deep spatial profiling (DSP), multiplex chromogenic immunohistochemistry, and confocal microscopy on human postmortem hippocampal samples, Fixemer et al. (2025) identified two spatially and molecularly distinct microglial AD populations: plaque-associated (PaM) and coffin-like (CoM) microglia. PaM are characterized by their rosette-like conformation around Aβ core plaques and exhibit molecular signatures enriched in complement system pathways (C1q, C3, C4b/d), ErbB signaling, lipid metabolism genes, PI3K-Akt signaling, and markers of necroptosis (pMLKL). PaM are surrounded by a reactive glial net (RGN) of polarized, hypertrophic astrocytes that express PCSK9, ITGA6, and C3. Additionally, CD163+ perivascular macrophages, including Iba1+CD163+ double-positive cells, are frequently incorporated into PaM, suggesting a contribution of infiltrating immune cells to the plaque microenvironment. In contrast, CoM are specifically enriched in the pyramidal layer of the CA2/CA1 hippocampal subfields, forming tightly packed, polarized cellular aggregates that often engulf neurons containing tau neurofibrillary tangles and phosphorylated α-synuclein inclusions. CoM display molecular signatures associated with protein degradation pathways, including STING, TGF-β signaling, NF-κB signaling, and ubiquitin-proteasome system (UPS) components such as SMURF2. CoM also express metabolic markers such as ACADS, suggesting high metabolic activity. Astrocytes surrounding CoM exhibit a dysmorphic phenotype with thin, irregular processes and lack of polarization toward the microglial aggregate. These findings underscore the heterogeneous roles of microglia in neurodegeneration, revealing that distinct microglial aggregates in the AD hippocampus are associated with different pathological protein aggregates, molecular pathways, and glial-immune microenvironments. Fixemer et al. (2025) highlights the complexity of glial-immune interactions in driving hippocampal degeneration in AD.

Beyond complement-mediated pruning, several signaling pathways directly regulate synaptic stability. The Wnt/β-catenin pathway maintains synaptic integrity by stabilizing β-catenin and inhibiting GSK3β, while the non-canonical Wnt-PCP pathway promotes synapse disassembly. Elliott et al. (2018) demonstrated that Aβ triggers Dkk1 expression, which shifts the balance from canonical Wnt/β-catenin (synapse-stabilizing) to non-canonical Wnt-PCP (synapse-destabilizing) signaling, thereby promoting synapse disassembly. Mechanistically, APP acts as a co-activator of both Wnt pathways through direct protein–protein interactions: the extracellular domain of APP binds to the Wnt-β-catenin co-receptor LRP6, while the intracellular YENPTY motif of APP binds to the Wnt-PCP co-receptor Vangl2. This interaction allows APP to bidirectionally modulate synapse stability. Importantly, Dkk1-induced synapse loss is APP-dependent, as APP-deficient neurons are protected against Dkk1-mediated dendritic spine withdrawal. Crucially, a pathogenic positive feedback loop was identified: Aβ induces Dkk1 expression, which activates Wnt-PCP signaling to promote synapse loss and, simultaneously, drives further Aβ production. Aβ production was positively correlated with Wnt-PCP activity and negatively correlated with canonical Wnt activity. This feedback loop represents a critical mechanism that links amyloid pathology to synaptic degeneration. Furthermore, the Swedish familial AD variant of APP (APPSwe) more readily co-activates non-canonical Wnt-PCP signaling at the expense of canonical Wnt activity, indicating that its pathogenicity involves direct effects on synapses beyond increased Aβ production.

APPSwe also exhibits altered trafficking consistent with enhanced amyloidogenic processing. The ROCK inhibitor fasudil can disrupt this positive feedback loop: by blocking Wnt-PCP activation, fasudil prevents Dkk1-induced synapse loss, and reduces Aβ production in primary neurons. *In vivo*, fasudil treatment for 14 days in 18-month-old 3xTg-AD mice significantly reduced both soluble and insoluble Aβ levels and decreases amyloid plaque burden across multiple brain regions, highlighting the therapeutic potential of targeting the Aβ-Dkk1-Wnt-PCP axis in AD (Elliott et al., 2018).

The therapeutic approaches that include ROCK inhibitors and Dkk1-neutralizing agents demonstrate potential to break the Aβ-mediated feedback mechanisms through their ability to block Wnt-PCP. The dual role of TGF-β in fibrosis and cancer development and its variable effects in AD patients makes it difficult to develop treatments that target specific pathways (Peng et al., 2022). Research needs to identify human-based models which show these communication pathways in order to develop successful treatments for AD disease progression.

The integrated pathways show how Aβ functions as a neurodegenerative trigger that also enhances the neurodegenerative process. Aβ-induced Dkk1 creates a positive feedback loop by upregulating Aβ synthesis in addition to promoting synaptic loss (Elliott et al., 2018). The two processes of oxidative stress and inflammation maintain a complex relationship through which ROS activate NF-κB to produce pro-inflammatory cytokines. IL-1β activates p38 MAPK, resulting in tau phosphorylation (Maurice and Angers, 2025; Mishra and Kumar, 2025). The Wnt/β-catenin pathway functions as a protective mechanism but becomes dysfunctional in AD, resulting in uncontrolled GSK3β activity that causes tau protein damage (Elliott et al., 2018). The PI3K/AKT signaling pathway that controls GSK3β activity becomes abnormal because of Aβ exposure, resulting in the breakdown of synaptic proteins (Dai et al., 2021; Alharthi et al., 2022). The signaling pathways need their spatial distribution to function properly.

P2RY12 receptors on microglial cells control blood vessel dimensions to establish a connection between vascular problems in AD, brain inflammation, and reduced blood flow (Mishra and Kumar, 2025). New tools are being utilized, including single-cell RNA-seq and microfluidic protocells, to study cellular interactions in high detail, while multi-omics methods identify new medical targets (Heuberger et al., 2025). However, research has not resolved all essential questions about TGF-β involvement in AD-related fibrosis development and its potential role in neuroinflammation control through strigolactones, which share signaling pathways with phytohormones (Peng et al., 2022; Ou et al., 2025). Current medical practices require interdisciplinary collaboration between structural and synthetic biologists to bridge the knowledge gaps that exist between available treatments and current medical practices for studying AD signaling pathways (Wang Z. et al., 2024; Heuberger et al., 2025).

The Wnt signaling system functions as an essential yet poorly understood pathway. It manages neurodegenerative disease progression through its ability to control tau protein phosphorylation in AD and tauopathies. Wnt signaling acts as a conserved pathway that controls cell division and proliferation through direct and indirect mechanisms to regulate tau phosphorylation. The Wnt/β-catenin signaling pathway develops abnormalities because of changes in vital kinases and phosphatases which result in abnormal tau hyperphosphorylation characteristic of AD. The Wnt signaling pathway regulates the central tau kinase GSK3β through its activity. The canonical Wnt signaling pathway leads to GSK3β inactivation by phosphorylating Ser9, resulting in decreased tau phosphorylation by GSK3β (Amal et al., 2019; Mueller et al., 2021; Stefanoska et al., 2022). The Wnt/Ca^2+^ pathway functions as a non-canonical Wnt pathway, leading to tau phosphorylation through the calcium-dependent activation of the CaMKII enzyme that shows increased activity in tauopathy models and causes synaptic damage (Amal et al., 2019; McKenney et al., 2025). In particular, the non-canonical Wnt/Ca^2+^ pathway is hyperactivated in tau transgenic mice models. The process of S-nitrosylation applies to RNF213 proteins, resulting in increased calcium release that leads to stronger NFAT-1 signaling activation. This cascade of events ultimately serves to exacerbate tau pathology (Amal et al., 2019).

#### 5.3.2 Selective neuronal vulnerability and resilience mechanisms

A hallmark of AD pathogenesis is the selective vulnerability of specific neuronal populations, particularly layer II neurons of the entorhinal cortex (EC), which exhibit early tau pathology and degeneration. Using single-nucleus RNA sequencing of postmortem human EC and superior frontal gyrus (SFG) across Braak stages 0, 2, and 6, Leng et al. (2021) identified RAR-related orphan receptor B (RORB) as a molecular marker of selectively vulnerable excitatory neurons in the EC. Specifically, EC excitatory neuron subpopulations expressing RORB (EC:Exc.s2 and EC:Exc.s4) showed a striking ∼50% decrease in relative abundance as early as Braak stage 2, with sustained depletion through stage 6. Notably, not all EC layer II neurons were vulnerable; other layer II-like subpopulations did not show significant changes, demonstrating that vulnerability is restricted to specific RORB+ subpopulations. Quantitative neuropathological validation using multiplex immunofluorescence confirmed that RORB+ excitatory neurons were preferentially depleted in EC superficial layers across Braak stages 0–6. Furthermore, phospho-tau inclusions detected by CP13 (targeting tau phosphorylated at Ser202) were significantly enriched in RORB+ neurons compared to RORB− excitatory neurons, supporting a model in which tau accumulation drives the selective loss of these neurons.

Transcriptomic analysis comparing RORB+ vulnerable neurons to all other EC excitatory neurons revealed distinct gene expression signatures. Genes with higher expression in vulnerable neurons were enriched for axon-localized proteins and voltage-gated potassium channels. Conversely, genes with lower expression in vulnerable neurons were enriched for synapse- and dendrite-localized proteins, as well as pathways involved in G-protein-mediated signaling, ion transport, and neurotransmitter receptor signaling. These molecular signatures may underlie their heightened susceptibility to tau pathology. Furthermore, a high expression of RELN was identified in vulnerable EC excitatory neurons, while its downstream effector DAB1 was broadly expressed across neuronal subtypes.

Immunohistochemistry has confirmed a significant reduction in Reelin-positive neurons in the EC of AD patients compared to controls. Independent validation in mouse models of AD revealed that both App-KI and Tau(P301S) mice exhibited decreased Reelin-positive neurons in the EC relative to wild-type controls, further supporting the association between Reelin expression and neuronal vulnerability. Importantly, the selective vulnerability of RORB-expressing neurons extended beyond the EC. In the SFG, RORB+ excitatory neuron subpopulations (SFG:Exc.s2 and SFG:Exc.s4) trended toward decreased relative abundance in Braak stage 6, consistent with the late appearance of tau pathology in the neocortex. Reanalysis of an independent prefrontal cortex dataset (Mathys et al., 2024) revealed that RORB+ excitatory neuron subpopulations (Mathys:Exc.s4) were significantly depleted in male AD patients, and these neocortical RORB+ neurons were transcriptionally most similar to vulnerable EC RORB+ neurons, suggesting shared mechanisms of vulnerability across brain regions.

In contrast to excitatory neurons, inhibitory neuron subpopulations in both EC and SFG showed no evidence of selective vulnerability in AD, consistent with their relative resistance to tau pathology. Additionally, an astrocyte subpopulation characterized by high GFAP expression (GFAPhigh astrocytes) was identified in both EC and SFG. These astrocytes upregulated reactive astrocyte markers (*CD44*, *HSPB1*, *TNC*, and *HSP90AA1*) and downregulated genes involved in glutamate/GABA homeostasis (*SLC1A2*, *SLC1A3*, *GLUL*, and *SLC6A11*) and synaptic adhesion/maintenance (*NRXN1*, *CADM2*, *PTN*, and *GPC5*), indicating a loss of homeostatic function that may contribute to neurodegeneration. The transcriptional signature of GFAPhigh astrocytes significantly overlapped with that of reactive astrocytes from a mouse model of spinal cord injury, and similar populations were identified in an independent prefrontal cortex dataset (Mathys et al., 2024). These findings provide a molecular framework for understanding selective neuronal vulnerability in AD, identify RORB as a key marker for vulnerable excitatory neurons, and implicate axon-localized proteins, potassium channels, and Reelin expression in modulating susceptibility to tau pathology (Leng et al., 2021).

Using single-nucleus RNA sequencing of 1.3 million cells from six brain regions across 48 individuals with and without AD, Mathys et al. (2024) constructed a multi-region transcriptome atlas of the aged human brain and identified five excitatory neuron subtypes that are disproportionately depleted in AD, including EC layer II RELN + lateral EC neurons, EC layer III RELN + neurons, EC layer V neurons, EC layer II/III TOX3+ TTC6+ neurons, and hippocampal CA1 pyramidal neurons. These vulnerable subtypes exhibited co-depletion patterns in AD individuals, particularly within established connections between CA1, subiculum, EC layer III, and EC layer V. Transcriptomic analysis revealed that vulnerable excitatory neurons share distinct gene expression signatures, including the enrichment of Reelin-signaling pathway components (*RELN* and *DAB1*), kinase-associated genes (*MAP2K5*, *PRKCA*, and *SPHKAP*), and genes involved in heparan sulfate proteoglycan biosynthesis (*HS6ST3*, *XYLT1*, and *NDST3*). Notably, Reelin-expressing excitatory neurons were preferentially depleted in the EC of AD patients by RNAscope, as well as in App-KI and Tau(P301S) mouse models. Furthermore, vulnerable inhibitory neuron subtypes in the prefrontal cortex were depleted in individuals with high tangle density. These inhibitory neurons similarly exhibited higher expression of Reelin signaling pathway genes (RELN, DAB1) and differential expression of Reelin receptors (*LRP8* and *NRP1*), suggesting shared mechanisms of vulnerability across neuronal classes. Furthermore, an astrocyte gene program associated with cognitive resilience to AD pathology was identified, linking choline metabolism (*PNPLA6*, *GPCPD1*, and *CHDH*) and polyamine biosynthesis (*ODC1*) to preserved cognitive function. This program was validated by RNAscope and confirmed in an independent cohort of 427 individuals (Mathys et al., 2024). These findings provide critical insights into why specific neuronal subpopulations degenerate preferentially in AD and identify Reelin signaling and heparan sulfate proteoglycan pathways as potential targets for therapeutic intervention.

Computational modeling approaches have enabled the quantitative investigation of microglial gene modulation in tau propagation dynamics. Anand et al. (2022) developed Nexis, a mathematical model that augments traditional network diffusion models by incorporating the effects of extra-connectomic molecular players, such as microglial gene expression, on tau accumulation and spread. Using this framework, they interrogated the modulatory effects of microglia on tauopathy progression in mouse models of tau seeding. The inclusion of Trem2 significantly improved the model’s predictive power compared to models without microglial influence or those incorporating microglial homeostasis genes (*P2ry12*, *Cx3cr1*, *Fcrls*, *Olfml3*, *Hexb*, *Siglech*, *Sox5*, and *Jun*). Trem2 exhibited dual and opposing effects on tau dynamics: it was associated with reduced intra-regional tau accumulation rate, while simultaneously increasing interregional tau spread rate from the hippocampal seed area to connected regions, including the striatum, pallidum, and contralateral hippocampus. Therefore, Trem2 may both mitigate local tau burden and facilitate its brain-wide propagation, reflecting opposing microglial functions in waste clearance versus disease propagation. Importantly, the effects of Trem2 were tau-strain-specific. In datasets DS6 and DS9 (characterized by tau from P301S mice and recombinant fibrils, respectively), Trem2 decreased accumulation but increased spread. In contrast, for DS4 (human AD brain-derived tau), Trem2 was associated with increases in both accumulation and spread, highlighting the heterogeneity of tau–microglia interactions.

Anand et al. (2022) also explored Apoe, another AD risk gene expressed in microglia and astrocytes, but they found that its inclusion did not significantly improve model fit, either alone or in combination with Trem2—consistent with Apoe acting downstream of Trem2 in the microglial response. Finally, *in silico* simulation of changes in Trem2 expression levels revealed a dose-dependent inverse relationship with tau pathology: higher Trem2 expression resulted in lower tau levels in selected brain regions, including the hippocampus, entorhinal cortex, neocortex, striatum, and amygdala, further supporting a protective role for Trem2 in tau accumulation. These modeling results provide the first quantitative characterization of microglial contribution to tau propagation at the whole-brain level and demonstrate that Trem2 modulates both tau accumulation and spread in a strain-specific manner, offering a framework for understanding the complex role of microglia in AD pathogenesis (Anand et al., 2022).

The scaffolding properties of tau create challenges for its interactions with Wnt signaling pathways. Tau’s intrinsically disordered structure allows it to act as a hub for kinases and phosphatases, which are also modulated by Wnt effectors (Mueller et al., 2021; McKenney et al., 2025). The pathogenic tau phosphorylation at Ser396/404 disrupts the essential interaction between tau and Fyn kinase, which controls NMDAR phosphorylation and leads to excitotoxicity. The disruption results in a reduction of synaptic strength (Regan et al., 2021). Wnt-mediated modifications in kinase activity may have an impact on this phosphorylation-dependent dissociation of tau–Fyn complexes. The redistribution of kinases triggered by Wnt signaling could disrupt the process by which tau protein keeps GSK3β molecules bound to microtubules, resulting in different rates of tau protein phosphorylation (Mueller et al., 2021; McKenney et al., 2025).

Tau proteins create a two-way communication system with Wnt proteins. Wnt signaling controls tau phosphorylation, but hyperphosphorylated tau proteins create a feedback loop which disrupts Wnt signaling pathways. The development of tauopathy models shows that tau species that cause disease prevent β-catenin breakdown through their disruption of β-TrCP1 ubiquitin ligase complexes, which results in uncontrolled Wnt/β-catenin signaling and neuron development into cancer stem cells (He et al., 2021; Han, 2025). The discovery of “master phosphorylation sites” that direct the tau phosphorylation cascade shows that p38α and other Wnt-regulated kinases should become the primary targets for therapeutic development (Stefanoska et al., 2022).

The drug interactions between these medications create major effects that impact medical treatment. The Wnt signaling pathway becomes dysregulated in tumors, leading to immune cell exclusion that may affect neuroinflammation in tauopathies through changes in microglial cell polarization patterns (Choi et al., 2024). Furthermore, prostate cancer models that are activated by the Wnt signaling pathway display elevated expression of ROR1, a receptor associated with non-canonical Wnt signaling. This receptor contributes to tau-induced synaptic damage, indicating that multiple neurological conditions have identical disease pathways (Choi et al., 2024). Future studies should establish whether tau–Wnt interaction creates immunotherapy resistance that affects cancer and neurodegenerative disease patients. Taken together, these results highlight the necessity of multi-target approaches that concurrently alter tau phosphorylation and Wnt signaling in order to break harmful feedback loops. Researchers should identify new interaction sites in complex networks through the combination of proximity-labeling proteomics with phospho-signature mapping, thus enabling the creation of specific treatment approaches for tauopathy (Prikas et al., 2022; Stefanoska et al., 2022).

The combination of Aβ and Wnt and inflammatory and oxidative stress pathways in AD demonstrates how the disease creates a complete body system that starts with local synaptic damage before it causes complete brain deterioration. Medical treatment requires multiple pathway targeting because shared effector molecules and feedback loops exist between different pathways. The research on AD pathogenesis has evolved through two recent discoveries that demonstrate microtubule-based signal transmission and mitochondrial information processing. AD pathogenesis extends past its established pathways because it uses signaling pathways that function through organelles (Picard and Shirihai, 2022; Choi et al., 2025). To confirm results from animal studies, future research should prioritize human-specific models, especially for pathways like IGF1R/INSR hybrids that display context-dependent behaviors (Sengupta and Mukhopadhyay, 2025). Research may determine the essential periods for intervention through an analysis of how these interactions progress through time and space from the initial appearance of oxidative damage to the final development of neuroinflammation. Application of this method to transform laboratory findings about disease mechanisms into medical practices may assist in managing or slowing AD progression.

### 5.4 Single-cell heterogeneity analysis

The advent of single-cell RNA sequencing (scRNA-seq) has profoundly transformed our comprehension of the selective vulnerability exhibited by distinct neuronal subpopulations in the early stages of AD pathogenesis. The main characteristic of AD involves the excessive deterioration of specific excitatory neurons, including those located in the entorhinal cortex, that show tau pathology and neuronal death during their initial stages (Leng et al., 2021). scRNA-seq technology was utilized to identify RORB as a specific molecular marker that exists only in the vulnerable excitatory neurons located in the entorhinal cortex. These neurons are progressively depleted throughout the course of AD and demonstrate a pronounced vulnerability to the formation of neurofibrillary tangle (Leng et al., 2021). ScRNA-seq enables researchers to identify molecular markers to reveal how different cell types express their genes according to their location in AD regions that show vulnerability. Studies have established vital knowledge about neuron vulnerability to disease-related damage resulting in early death. The vulnerable neurons display specific gene expression patterns that interfere with Reelin signaling and heparan sulfate proteoglycan biosynthesis to increase their risk of tau accumulation (Mathys et al., 2024).

ScRNA-seq technology enables researchers to study cellular diversity within brain tissues at a previously impossible level of detail. Five excitatory neuron subtypes, such as entorhinal cortex layer II and hippocampal CA1 neurons, are disproportionately diminished in AD patients, according to a multi-region transcriptome atlas of the older adult human brain, further suggesting region-specific vulnerability (Mathys et al., 2024). The neurons show identical genetic patterns that connect to synaptic plasticity and metabolic stress pathways to indicate that degeneration occurs through identical pathways (Mathys et al., 2024). The scRNA-seq technique showed that reactive astrocytes exist as multiple subgroups which express homeostatic genes at lower levels to weaken neuroprotective function and increase neuronal vulnerability to damage (Leng et al., 2021). Furthermore, scRNA-seq technology performs two functions: identifying susceptible cells and demonstrating how non-cell-autonomous mechanisms contribute to AD progression.

scRNA-seq data have been utilized to understand selective vulnerability in combination with additional omics information. The analysis of whole-genome sequencing data with snRNA-seq results has shown that microglial subtypes function through independent control mechanisms that affect both peripheral immune responses and that genes that increase the risk of AD lead to oligodendrocyte damage (Wang Q. et al., 2024). The multi-omics techniques demonstrate that AD develops as a body-wide process that causes specific transcriptional changes in different cell types, which may indicate broader network disruptions. The scRNA-seq analysis detected two separate astrocyte groups that expressed their genes differently for choline metabolism and polyamine production. These two pathways were regarded as potential therapeutic approaches to boost cognitive resistance against AD progression (Mathys et al., 2024). ScRNA-seq technology has been employed to create new medical solutions to protect neurons better or modify glial cell functions by understanding their unique molecular patterns.

However, current scRNA-seq data processing for therapeutic applications faces new challenges due to recent scientific discoveries. The research methods used in nuclear RNA-seq studies fail to detect all microglial activation states and specific changes that occur in the local transcriptome (Mathys et al., 2024). The different characteristics of AD patients require larger cohorts containing a variety of patient types to confirm cell-type-specific signatures in different population groups. Future research should utilize spatial transcriptomics to identify which neurons are most at risk in their original brain structure, and CRISPR-based assays should be used to verify the functional role of genes identified as potential candidates (Sanchís-Calleja et al., 2025). ScRNA-seq has revealed the molecular factors that make neurons susceptible to AD while creating new treatment approaches that focus on treating the disease at its initial stages (Table 2).

**TABLE 2:** Key pathways and gene targets identified in AD brain organoid studies.

Pathway/Gene target	Organoid model	Key finding	Translational potential	Refs
Wnt/β-catenin signaling	iPSC-derived cerebral organoids from PSEN1 E280A carriers with APOE3 Christchurch variant	APOE3Ch enhances Wnt/β-catenin signaling, reduces tau phosphorylation, and protects against PSEN1 mutation-induced pathology	Wnt pathway activators or APOE3Ch mimetics could serve as therapeutic agents for AD and tauopathies	Perez-Corredor et al. (2024)
*LINGO2*	Xenografted human iPSC-derived neurons with APP V717I mutation	LINGO2 identified as upregulated gene in AD neurons; knockdown rescues neurite outgrowth deficits and reverses AD-associated transcriptional changes	LINGO2 inhibition represents a potential therapeutic strategy for synaptic dysfunction in AD	Qu et al. (2024)
PI3K/Akt pathway	SH-SY5Y neuronal models overexpressing APP	APP overexpression protects mitochondria from rotenone-induced oxidative damage through PI3K/Akt pathway activation	PI3K/Akt activators could provide neuroprotection in mitochondrial dysfunction-related neurodegeneration	Cimdins et al. (2019)
β-catenin signaling	Human neural stem cells (hNS1) with APP knockdown	APP reduction shifts differentiation toward neurogenesis and decreases gliogenesis via β-catenin signaling	Targeting APP-β-catenin interaction could modulate neural stem cell fate in regenerative approaches	Coronel et al. (2023)
*TREM2/APOE*	Forebrain organoids with isogenic microglia-like cells	Microglia modulate neuroinflammation via EV-mediated communication; differential expression of TREM2 and CASS4 in response to AD EVs	EV-based therapies or TREM2 modulators could target neuroinflammation in AD	Liu et al. (2025a)
Synaptic function-related genes	Cerebral organoids integrated with microglia-like cells	Microglia promote neuronal maturation, synaptic function, and network activity; increased gamma-band oscillations	Targeting microglia-neuron interactions could enhance synaptic health	Fagerlund et al. (2021)
Tight junction proteins (CLDN5 and ZO-1)	Vascularized brain organoids with endothelial networks	Formation of functional neurovascular units with tight junction proteins; reduced core necrosis	Enhancing BBB integrity through tight junction stabilization could ameliorate vascular dysfunction in AD	Sun et al., 2022; Fumadó Navarro et al., 2025
VE-cadherin, VCAM-1	3D blood–brain barrier neurosphere co-culture model	Aβ oligomers induce endothelial barrier disruption and pericyte loss; elevated soluble Aβ40 levels	Targeting endothelial dysfunction and vascular inflammation could address vascular contributions to AD	Ko et al. (2023)
Multiple AD-related pathways	3D brain spheroids from AD patient iPSCs	Aβ accumulation; caspase-induced cell death; platform for logical network-based drug screening	Network-based drug screening identifies patient-specific drug responses; enables precision medicine	Park et al. (2021)
Multiple AD pathologies	Vascularized neuroimmune organoids exposed to sporadic AD brain extracts	Development of tau tangles, Aβ plaques, neuroinflammation, and synapse loss within 4 weeks	Platform for testing immunotherapies and studying sporadic AD mechanisms	Ji et al. (2025)
APP processing pathways	Cerebral organoids from Dutch-type CAA patients (*APP* E693Q mutation)	Rapid Aβ40 deposition; vascular amyloid pathology; disrupted TGF-β signaling	Targeting APP processing and TGF-β signaling could address vascular amyloid pathology	Daoutsali et al. (2023)
APOE ε4-related pathways	Directly reprogrammed 3D brain organoids from APOE ε4 fibroblasts	Accelerated Aβ42 accumulation; gene expression profiles matching AD patient brains	APOE ε4 correction or downstream pathway modulation represents therapeutic opportunity	Kim et al. (2024)
Serum-induced pathways	iPSC-derived brain organoids exposed to human serum	Aβ aggregation, tau phosphorylation, synaptic dysfunction; BACE and GSK3α/β upregulation	Targeting BACE1 and GSK3α/β could address sporadic AD mechanisms	Chen et al. (2021a)
Wnt/β-catenin, cadherin signaling	iPSC-derived cerebral organoids from *PSEN1* E280A carriers	scRNA-seq revealed regulation of cadherin and Wnt signaling pathways by APOE3Ch	Wnt pathway modulators could serve as therapeutic targets	Perez-Corredor et al. (2024)

### 5.5 Environmental and metabolic risk factors in AD pathogenesis

Emerging evidence highlights the significant role of environmental and metabolic risk factors in the onset and progression of AD, with brain organoid models providing unprecedented insights into the underlying mechanisms (Liu B. et al., 2025).

#### 5.5.1 Cholesterol imbalance and lipid dyshomeostasis

The APOE ε4 genotype is the most prevalent genetic risk factor for late-onset AD, and its impact on lipid metabolism has been extensively studied in organoid models. Nemergut et al. (2023) demonstrated that the C112R substitution in ApoE4 triggers long-distance conformational changes, resulting in a V-shaped dimeric unit that is geometrically distinct from the T-shaped unit preferred by ApoE3. This structural difference, mediated by the self-association interface involving residues, including W34, R38, E45, E49, and R145, renders ApoE4 more prone to aggregation. The treatment of APOE ε4/ε4 cerebral organoids with the drug candidate tramiprosate, metabolized to 3-sulfopropanoic acid (SPA), revealed specific effects on cholesteryl esters (particularly the upregulation of polyunsaturated species CE18:2, CE18:3, CE22:4, and CE24:4) alongside the downregulation of carnitines and ether-linked phospholipids. Mechanistically, SPA induces ApoE3-like conformational behavior in ApoE4 through non-specific interactions with charged surface residues, modulating both aggregation propensity and lipid homeostasis (Nemergut et al., 2023). Zhao et al. (2021) demonstrated that APOE deletion in human iPSC-derived cerebral organoids leads to the increased accumulation of insoluble α-synuclein and phosphorylated α-synuclein, accompanied by lipid droplet accumulation, altered fatty acid utilization, and elevated cholesterol ester levels at Day 90 of differentiation. These phenotypes were associated with reduced GBA expression and activity, as well as endolysosomal dysregulation. Importantly, treatment with exogenous astrocyte-derived apoE2 and apoE3 partially rescued the accumulation of insoluble α-synuclein and lipid droplet formation in APOE-deficient cerebral organoids, whereas apoE4 failed to exert similar protective effects. Consistent with these findings, cerebral organoids from APOE4 homozygous donors exhibited higher levels of insoluble α-synuclein than those from APOE3 homozygous donors, and postmortem brains from Lewy body disease patients carrying APOE4 showed increased apoE co-localization with Lewy bodies (Zhao et al., 2021). Glasauer et al. (2022) demonstrated that in human cerebral organoids carrying pathogenic MAPT mutations (V337M and R406W), single-cell RNA sequencing revealed the cholesterol biosynthesis pathway in astrocytes as the most significantly upregulated gene set compared to isogenic controls. The upregulated genes included key regulators of cholesterol metabolism: *HMGCR* (rate-limiting enzyme), *ACAT2* (cholesterol esterification), *LDLR* (cholesterol uptake), and *SREBF2*. Lipidomics analysis confirmed elevated levels of cholesterol and its precursors (desmosterol, 7-DHC, and 8-DHC) in mutant organoids at 7 months, with metabolic changes lagging behind transcriptional upregulation. These findings suggest that cholesterol dyshomeostasis in astrocytes represents an early event in the etiology of tauopathy-related neurodegeneration, preceding overt pathology and neuronal loss (Glasauer et al., 2022).

#### 5.5.2 Mitochondrial dysfunction

Mitochondrial dysfunction is a hallmark of AD, and iPSC-derived neuron models have enabled the detailed characterization of these deficits across different genetic forms of the disease. MacMullen et al. (2025) designed a high-throughput longitudinal phenotypic assay to track mitochondrial dynamics and bioenergetics in iPSC-derived glutamatergic neurons carrying mutations in PSEN1, PSEN2, and APP. Mutations in all three genes altered mitochondrial function, including basal, ATP-linked, and maximal oxygen consumption rates, as well as spare respiratory capacity, with PSEN1/PSEN2 alleles showing more severe phenotypes than APP mutations. Notably, mitochondrial fragmentation and neurite degeneration were dramatic in PSEN1/PSEN2 alleles but essentially absent in APP alleles, suggesting distinct mechanisms of mitochondrial pathology across different familial AD forms (MacMullen et al., 2025). Whitehouse et al. (2025) utilized a novel 3D bioprinted forebrain cortex model that incorporated glutamatergic neurons, GABAergic neurons, astrocytes, and microglia to investigate the mechanisms that underlie impaired neurite outgrowth in AD. Using iPSC-derived neural progenitor cells carrying APP mutations (K670N/M671L + V717F), they demonstrated that APP mutant cultures exhibited significantly reduced mitochondrial membrane potential and fragmented mitochondrial networks compared to isogenic controls. These mitochondrial abnormalities, indicative of dysfunction and potential cellular energy deficits, were accompanied by elevated oxidative stress and impaired mitophagy. Notably, these pathological features were more pronounced in 3D than in 2D cultures, correlating with fragmented, shorter neurites and reduced branching complexity, suggesting that mitochondrial dysfunction may limit neurite extension in AD.

#### 5.5.3 Neuroinflammation and microglial responses

The integration of microglia into brain organoid models has revolutionized our understanding of neuroinflammation in AD. Rao et al. (2025) synthesized recent advances in human-based models—including stem cell-derived microglia, cerebral organoids, assembloids, and human–mouse chimeric systems—that revealed human-specific microglial responses to amyloid plaques and their regulation of neuroinflammation, which are not recapitulated in traditional animal models. Their review highlights how single-cell technologies applied to *post* postmortem human brain tissue and iPSC-derived models have uncovered distinct microglial transcriptional states associated with AD progression, including disease-associated microglia (DAM) signatures, lipid droplet-accumulating microglia linked to APOE4/4 genotype, and CD83^+^ microglial subtypes correlated with increased plaque and tangle pathology. These human models provide unprecedented insights into the multifaceted roles of microglia in AD and open new avenues for therapeutic interventions that target microglial function (Rao et al., 2025).

Liu C. et al. (2025) developed isogenic forebrain cortical organoids (iFCo) co-cultured with microglia-like cells (MGCs) derived from the same human iPSC line, creating an immune-competent brain model. Stimulation with extracellular vesicles (EVs) from AD-patient-derived brain organoids carrying APOE ε4/ε4 or *PSEN1* mutations induced differential responses: while MGCs alone showed an upregulation of pro-inflammatory genes (IL-6, IL-12β, iNOS, TNFα), the co-cultured organoids exhibited mitigated inflammatory responses, thus demonstrating the regulatory role of the neural microenvironment. Treatment with EVs from healthy MGCs (both unstimulated M0 and anti-inflammatory M2 phenotypes) or from unstimulated co-cultures significantly reduced the expression of pro-inflammatory genes IL-12β and iNOS, as well as AD risk genes TREM2 and CASS4, in APOE4 EV-stimulated co-cultures. Proteomic and miRNA sequencing of AD EVs revealed an enrichment of APOE and APP proteins and miRNAs targeting pathways, including mitophagy. Liu C. et al. (2025) establish a platform for modeling microglia-neuron interactions in AD and demonstrate the therapeutic potential of EVs derived from healthy MGCs and their co-cultures. Becerra-Calixto et al. (2025) developed a neuroimmune assembloid model by integrating cerebral organoids (COs) with induced microglia-like cells (iMGs) derived from the same familial AD (fAD) patient iPSC line carrying a PSEN2 mutation (N141I). After 120 days in co-culture, these assembloids recapitulated key histopathological and immunological features of AD, including amyloid plaque-like structures (detected by 6E10 immunoreactivity) and neurofibrillary tangle-like structures (p-Tau Ser396-positive). fAD iMGs within the assembloids exhibited an activated pro-inflammatory phenotype characterized by upregulated TREM2, downregulated P2RY12, increased IL-6 production, and significantly reduced phagocytic capability for Aβ peptides than in healthy controls. Transcriptomic analysis revealed an enhanced expression of genes associated with neuroinflammation, apoptosis, and microglial activation in fAD assembloids, as well as autophagosome-like structures with increased LC3B and ubiquitin positivity. This model provides a robust platform for investigating microglia–neuron interactions in AD pathophysiology and testing the therapeutic strategies that target neuroinflammation (Becerra-Calixto et al., 2025).

#### 5.5.4 Environmental exposure

Beyond metabolic factors, emerging evidence has implicated environmental pollutants in AD pathogenesis. Ahmed et al. (2025) synthesized mechanistic studies from the past decade on the associations between exposure to four heavy metals—arsenic (As), manganese (Mn), lead (Pb), and cadmium (Cd)—and AD pathogenesis. These metal exposures induce a range of pathological processes that intersect with established AD mechanisms, including oxidative stress, mitochondrial dysfunction, protein aggregation (Aβ and tau), neuroinflammation, autophagy dysfunction, and tau hyperphosphorylation. While certain pathways are shared across metals, the review also highlights metal-specific effects: Pb exposure disrupts the BBB and epigenetically alters AD-related gene expression through DNA methylation and histone modification; Cd triggers neuronal senescence via activation of the p53/p21/Rb pathway; As disrupts nitric oxide (NO) signaling and cortical synaptic function; Mn induces glutamate excitotoxicity and dopaminergic neuron damage. Ahmed et al. (2025) provide a framework for understanding metal-specific contributions to AD and identify potential targets for therapeutic intervention.

Liu B. et al. (2025) synthesized recent evidence on the impact of environmental pollutants on AD pathology. Prolonged exposure to particulate matter (PM2.5), heavy metals (including lead, cadmium, mercury, and arsenic), and engineered nanomaterials (such as silver, iron oxide, and silica) significantly increases AD risk. These environmental exposures converge on key pathological mechanisms, including oxidative stress, neuroinflammation, mitochondrial dysfunction, and disruption of the BBB and ultimately drive amyloid-β plaque deposition and tau hyperphosphorylation. Liu B. et al. (2025) also discuss the synergistic effects of combined pollutant exposures and emphasize the need for improved human-relevant models, standardized exposure assessment protocols, and public health strategies to mitigate environmental contributions to AD. These organoid-based studies collectively demonstrate that environmental and metabolic risk factors converge on common pathological pathways in AD, providing new opportunities for therapeutic intervention and underscoring the importance of human-relevant models in understanding disease mechanisms.

### 5.6 Comparative modeling of familial and sporadic Alzheimer’s disease using brain organoids

The vast majority of AD cases are sporadic (sAD), accounting for >99% of all patients, with a typical onset age of over 65 years. In contrast, familial AD (fAD) comprises approximately 1% of cases, is caused by autosomal dominant mutations in APP, PSEN1, or PSEN2, and typically presents before age 65 (Gonzalez et al., 2018; Humpel, 2025). Brain organoid technology has enabled remarkable advances in modeling AD pathology. In this study, cerebral organoids were generated from human-induced pluripotent stem cells (iPSCs) derived from patients with familial AD (fAD, carrying the PSEN1 A246E mutation) and DS. These organoids spontaneously develop Aβ plaques and phosphorylated tau aggregates, faithfully recapitulating the two core pathological features of AD. In contrast, current organoid models are primarily used to study hereditary AD, whereas modeling sporadic AD (sAD)—which accounts for the vast majority of cases—remains challenging due to the lack of clear disease-causing gene mutations. This situation highlights the need to develop sAD organoid models that integrate multiple risk factors (such as APOE4, vascular damage, and inflammation) to improve the translational relevance of drug discovery, as many anti-Aβ therapeutics developed based on insights from hereditary AD have shown limited efficacy in sporadic patients (Abushakra et al., 2025).

#### 5.6.1 Distinct pathogenic timelines and phenotype induction

Cerebral organoids (COs) derived from iPSCs that carry familial AD mutations in PSEN1 or APP spontaneously develop AD-like pathological features within 1.5–6 months in culture. Among these features, an elevated Aβ42/Aβ40 ratio becomes significant at 5–6 months. Monomeric phosphorylated tau (p-Tau, including pT217 and pT181) can be detected as early as 1.5 months, whereas high-molecular-weight oligomeric species gradually accumulate between 3 and 6 months, exhibiting a time-dependent pathological evolution (Gonzalez et al., 2018; Labra et al., 2026). For example, cortical organoids carrying the APPSwe/WT mutation exhibit a time-progressive loss of monomeric tau, while both PSEN1ΔE9/WT and APPSwe/WT organoids accumulate aggregated high-molecular-weight p-Tau species (pT181 and pT217) within 3–6 months of differentiation (Arber et al., 2021; Labra et al., 2026). Notably, fAD mutations in PSEN1 also lead to premature neurogenesis—driven by reduced Notch signaling—which is detectable even at early differentiation stages and is recapitulated in both 2D cortical cultures and 3D cerebral organoids.

In contrast to fAD organoids that carry deterministic genetic mutations, sAD organoids do not develop spontaneous pathology without exogenous triggers as they lack disease-causing mutations. To model sAD, researchers can introduce environmental or metabolic risk factors that mimic the multifactorial etiology of the disease. One effective approach is serum exposure: treating hiPSC-derived brain organoids with human serum (from aged individuals or AD patients) induces Aβ aggregation, tau phosphorylation, synaptic loss, impaired neural network activity, and astrocytic immune responses, thereby recapitulating key pathological features of sAD (Chen X. et al., 2021). This method models BBB leakage—a known sAD risk factor—and reproduces the complex interplay of blood-derived factors that contribute to sAD pathogenesis.

#### 5.6.2 The central role of the APOE genotype in sAD modeling

APOE ε4 is a strong genetic risk factor for late-onset AD and dementia with Lewy bodies. Unlike familial AD mutations, APOE does not directly alter APP processing or γ-secretase activity; instead, in iPSC-derived cerebral organoid models, APOE regulates lipid metabolism, cholesterol homeostasis, GBA activity, and endolysosomal trafficking, thereby significantly affecting α-synuclein aggregation and pathology. APOE deficiency or the expression of APOE4 exacerbates insoluble α-synuclein accumulation and lipid droplet formation, whereas APOE2 and APOE3 exert protective effects. These findings reveal the lipid metabolism-related mechanisms by which APOE4 drives synucleinopathies (Zhao et al., 2021). Brain organoids have proven valuable for dissecting APOE genotype-specific effects. Zhao et al. (2021) showed that APOE4 cerebral organoids exhibit increased accumulation of insoluble α-synuclein, along with altered lipid metabolism and endolysosomal dysregulation. Separately, Nemergut et al. (2023) demonstrated that the drug candidate tramiprosate (metabolized to SPA) induces APOE3-like conformational behavior in APOE4 through interactions with charged surface residues, reducing APOE4 aggregation and modulating lipid homeostasis. These findings are particularly relevant for sAD drug development. In a recent phase III trial (APOLLOE4) of valiltramiprosate (ALZ-801) in APOE4/4 homozygotes with early AD, the overall population did not achieve significant clinical efficacy on the primary endpoint (ADAS-Cog13). However, the trial showed a significant slowing of hippocampal atrophy in the overall population. In the prespecified mild cognitive impairment subgroup, valiltramiprosate demonstrated nominally significant clinical benefits on ADAS-Cog13, along with positive trends on functional outcomes (Abushakra et al., 2025; Hey et al., 2025).

#### 5.6.3 Non-genetic and environmental factors in sAD

Han et al. (2024) used a human embryonic stem cell-derived 3D cortical organoid model to simulate early human brain development and investigate the neurotoxic mechanisms of fine particulate matter (PM2.5). Their results showed that exposure to 5 μg/mL and 50 μg/mL PM2.5 induced neuronal apoptosis, disrupted neural differentiation, and caused abnormal expression of cortical layer markers in the organoids. Transcriptomic and pathway enrichment analyses further revealed that PM2.5 primarily caused mitochondrial complex I dysfunction and axon guidance pathway disturbances, molecular changes that closely resemble the pathogenesis of Parkinson’s disease, rather than directly inducing beta-amyloid (Aβ) deposition or tau hyperphosphorylation. Therefore, Han et al. (2024) provide human-relevant model evidence that PM2.5 increases the risk of Parkinson’s disease through developmental neurotoxicity pathways but does not directly demonstrate that PM2.5 induces AD-like pathology in organoids. Korde and Humpel (2024) used hippocampal brain slices from postnatal day 8–10 mice (organotypic brain slices, and not organoids). They locally delivered human Aβ42 and P301S aggregated tau via collagen hydrogels, combined with nanomolar concentrations of heavy metals (aluminum, lead, and cadmium) or intracellular pathway modulators (scopolamine, wortmannin, and MHY1485) added to the culture medium. Their results showed that heavy metal treatment significantly enhanced Aβ plaque-like pathology, with Aβ preferentially accumulating inside neurons and subsequently leading to cell death. In contrast, the pathway modulators primarily promoted tau neurofibrillary tangle-like pathology. Combining lead and cadmium with the three pathway modulators simultaneously induced both core pathological features of AD: Aβ and tau pathologies. Although this model is an *ex vivo* system reliant on exogenous protein addition and pharmacological intervention, it provides a rapid, cost-effective platform suitable for drug screening to study AD-like pathologies and reveals the potential synergistic contribution of environmental heavy metals and dysregulated intracellular signaling pathways to the pathogenesis of AD (Korde and Humpel, 2024). These models demonstrate that environmental insults can trigger AD-like pathology in the absence of fAD mutations, providing a platform for studying sporadic disease mechanisms and testing preventive strategies.

#### 5.6.4 Cross-validation between fAD and sAD models

In fAD patient-derived cerebrocortical organoids carrying PSEN1 or APP mutations, key AD pathologies are recapitulated, including elevated Aβ42/40 ratio, progressive accumulation of high-molecular-weight phosphorylated tau (p-Tau217 and p-Tau181), synaptic loss, and neuronal hyperexcitability. Transcriptomic and functional analyses reveal impaired autophagy, mitochondrial dysfunction, and altered excitatory/inhibitory neuronal balance. These phenotypes are partially rescued by chronic treatment with the lysosomal flux activator CCT020312, which reduces p-Tau oligomers and restores synaptic proteins (Labra et al., 2026). Advances in iPSC technology have enabled the modeling of both fAD and sAD. fAD iPSC-derived neurons and organoids (carrying PSEN1 or APP mutations) consistently recapitulate increased Aβ42/40 ratios and elevated p-Tau, though extracellular plaques and tangles are rarely observed. sAD iPSC models, particularly those with APOE4 genotype, exhibit distinct phenotypes, including dysregulated cholesterol/lipid metabolism, impaired Aβ uptake by astrocytes, and neuroinflammatory gene expression changes. While both fAD and sAD models show synaptic dysfunction and organelle pathology, the molecular drivers and onset kinetics differ, reflecting the heterogeneity of AD. However, direct comparative transcriptomic analyses between fAD and sAD organoids are currently limited, and no single study has yet systematically contrasted their pathway enrichments (Sahlgren Bendtsen and Hall, 2023). The development of isogenic controls through CRISPR/Cas9 editing enables the direct comparison of mutations versus genotype-specific effects—for example, introducing fAD mutations into APOE ε4 background organoids to model gene–gene interactions.

#### 5.6.5 Translational implications and the fAD-to-sAD gap

The clinical reality is that many anti-Aβ therapeutics have historically failed in clinical trials. However, lecanemab and donanemab are the first monoclonal antibodies with unequivocal evidence of reducing cognitive and functional decline in AD, albeit with modest effect sizes. Their approval has sparked debate regarding the benefit-to-risk ratio, particularly due to amyloid-related imaging abnormalities (ARIA) in a minority of patients, as well as significant cost and accessibility concerns. Only a small proportion of patients with early AD in real-world settings would qualify for treatment under strict trial eligibility criteria, raising questions about generalizability and health-system capacity (Frisoni et al., 2025a). In the phase III APOLLOE4 trial, valiltramiprosate did not show significant clinical efficacy in the overall early AD population at 78 weeks, despite slowing hippocampal atrophy (18%). Prespecified analyses in the MCI subgroup showed nominally significant cognitive and functional benefits, highlighting the potential importance of earlier intervention (Abushakra et al., 2025). This underscores that organoid models—whether fAD or sAD—must be critically evaluated for their predictive validity. Future research should prioritize (i) developing sAD organoid models that incorporate APOE ε4 genotype and physiological aging cues, (ii) validating drug candidates in both fAD and sAD organoid platforms before clinical trials, and (iii) using multi-omics integration to identify which fAD-derived mechanistic insights actually translate to sAD.

#### 5.6.6 Outlook

The field is gradually shifting from fAD-dominant models toward more representative sporadic late-onset AD (LOAD) models that incorporate genetic susceptibility (e.g., APOE4) and aging. Sun Z. et al. (2024) demonstrated that directly reprogrammed neurons from LOAD patient fibroblasts, cultured in a 3D spheroid environment, recapitulate key neuropathological features, including Aβ deposition, tauopathy, and spontaneous neurodegeneration—phenotypes absent in iPSC-derived neurons due to the erasure of donor age-related signatures. Aging is a major risk factor for late-onset neurodegenerative diseases. While iPSC-based neuronal models reset donor age to an embryonic-like state, directly reprogrammed neurons (iNs) retain critical aging hallmarks such as epigenetic age, mitochondrial dysfunction, telomere shortening, and impaired proteostasis. This preservation of age-related signatures makes iNs a more suitable platform for modeling late-onset diseases like LOAD than iPSC-derived neurons which often fail to exhibit overt disease phenotypes (Aversano et al., 2022). However, the comparative limitations of each model must be transparently reported: fAD organoids offer deterministic, reproducible pathology but may not capture sporadic disease heterogeneity; sAD organoids are more clinically relevant but require complex induction protocols and show greater variability. A combined approach—using isogenic series to dissect genetic contributions while validating findings in sAD models—represents the current best practice for organoid-based AD research.

## 6 Application of organoids in AD drug screening

### 6.1 Establishment of high-throughput screening platforms

By utilizing the special qualities of microfluidic platforms for sensitive, quick, and compact biochemical investigations, the use of microfluidic chip technology in AD research and diagnosis is a developing field. The development of diagnostic tools for complicated disorders such as AD requires microfluidic platforms to achieve the downsizing, integration, and automation of biochemical assays (Haeberle and Zengerle, 2007). The platforms enable users to perform different microfluidic operations that can be merged into customized assays for specific applications while providing flexible operation and potential for single-piece integration.

The field of microfluidics has expanded its research applications in biomedical science through three new developments: flow cytometry, surface-enhanced Raman scattering (SERS), and tissue-based bioassays. The new developments in microfluidics technology enable AD research to be conducted at higher levels. The creation of miniaturized flow cytometry systems, which operate on chip platforms, fulfills clinical requirements because they perform fast cellular tests which can be adapted to identify AD-related cellular biomarkers (Chung and Kim, 2007). Highly sensitive chemical and biological detection systems have been established through the combination of SERS, with microfluidic platforms that enable the detection of small amounts of disease-related substances (Chen and Choo, 2008).

The development of organ-on-a-chip systems serves as a demonstration of how microfluidic technology enables the creation of human tissue and disease process simulations. These systems enable AD disease mechanisms and new treatments to be tested through controlled experiments that duplicate the natural conditions of brain tissue environments (Li et al., 2012; Wu et al., 2020). The biological value of these models thus increases because researchers can grow cells in three-dimensional structures inside microfluidic devices, which helps to determine drug responses and disease processes. The exact reproduction of brain tissue environments through microfluidic chip systems makes this technology a revolutionary scientific tool for studying neurodegenerative diseases, including AD.

The human brain contains intricate 3D structures that differ from the structure of conventional 2D cell cultures and animal models that fail to produce effective clinical results (Liu et al., 2022; Shaner et al., 2023). Brain-on-a-chip systems based on microfluidics monitor neural network activities, synaptic breakdown, and Aβ plaque formation in real time using neural stem cells or organoids within biomimetic microenvironments (Lefebvre et al., 2024). The platforms enable budget-friendly drug screening and mechanistic studies, linking *in vitro* testing to *in vivo* models.

The creation of 3D NSC spheroid-based biochips with MEAs is a key advance in AD research. Impedance tests to monitor Aβ-induced neurotoxic effects can be implemented with the development of brain-like structures through neuronal growth and network development (Carstens et al., 2025). The results from immunostaining and scanning electron microscopy experiments show that Aβ proteins accumulate near-neurites that match results observed in living tissues (Pedrero-Prieto et al., 2019). The combination of MEAs which monitor network breakdown produces real-time output that matches biological measurements (Sato et al., 2023; Hua et al., 2025). The method produces better results than static 2D models because it allows the monitoring of neurodegeneration progression across time and space, which is vital for creating new medical interventions.

BBB models using microfluidic chip technology have been developed. Three disease-related characteristics use pre-differentiated AD neurospheres with a 3D self-assembled BBB vascular network to detect elevated soluble Aβ40 levels and dystrophic neurites (Ko et al., 2023; Liu T. et al., 2025). The chip’s BBB integrity was damaged during Aβ treatment, demonstrating that oxidative stress in clinical AD patients causes blood vessels to become more permeable. The dual-compartment design allows investigation into neurovascular interaction to identify crucial elements for AD progression. Future versions will incorporate patient-derived organoids together with continuous-flow systems to achieve better physiological accuracy, while showing how BBB breakdown results in Aβ plaque formation.

Microfluidic chips are increasingly used for AD-related protein analysis and biomarker identification in addition to disease modeling. The protein aggregations that appear in AD pathology can be predicted through 2D native protein electrophoresis tests that run on microfluidic devices (Yu et al., 2016). The method demonstrates that microfluidic electrophoresis operates as a highly sensitive diagnostic system that enables the detection of diseases at their beginning stages and tracks their development.

Microfluidic chip technology has revolutionized AD biomarker identification and evaluation through its superior sensitivity, cost-effectiveness, and ability to scale up operations. The neurodegenerative disease AD advances through the development of Aβ plaques and hyperphosphorylated tau proteins, which serve as vital markers for both initial diagnosis and assessing follow-up treatment (Surpi et al., 2023; Wang Y.-T. et al., 2024). Current diagnostic methods include PET scans and CSF tests to provide accurate results; however, their implementation faces challenges due to high expense, invasive procedures, and restricted availability (Liu et al., 2018; Wang Y.-T. et al., 2024). Current detection problems are solved through microfluidic platforms that achieve size reduction and automated biomarker detection processes that produce high analytical results. The microfluidic system proved its ability to extract Aβ1–42 from medical samples through its magnetic nanoparticle and hyperbranched KVLFF aptamer components, which generated a 50-nL plug that contained 90% of all Aβ1–42 molecules for better detection results (Surpi et al., 2023). The system operates through permanent magnets, which produce strong magnetic fields to achieve both biomarker management and early AD detection capabilities (Surpi et al., 2023).

The development of label-free low-cost sensors for Aβ aggregation monitoring in AD has become possible through microfluidic design innovations, enabling real-time Aβ aggregation detection. The platform enables users to access disposable point-of-care compatible tools to track oligomerization in real time for studying AD progression and developing new aggregation inhibitors (Liu et al., 2018). The study established an Aβ oligomer detection limit of 23.7 pg/mL through the combination of wax-printed multi-chamber paper devices with copper-enhanced gold nanoprobe colorimetric immunoblotting and shows potential for developing affordable diagnostic methods that do not require specialized equipment (Phan and Cho, 2021). The development of microfluidics technology enables researchers to modify different detection methods, including electrochemical and optical and magnetic systems, for medical applications.

The combination of microfluidics with NULISATM advanced proteomic technology enables biomarker analysis to operate in previously inaccessible areas. The NULISA platform enables the detection of 120 CNS disease-related proteins at high sensitivity through its use of oligonucleotide-conjugated antibodies and dual capture-release technology, which minimizes background interference (Wang Y.-T. et al., 2024; Yeon et al., 2025). The need for thorough biomarker panels in differential diagnosis may be met by using such assays in conjunction with microfluidics to enable high-throughput, automated profiling of AD biomarkers from small sample volumes (Li Y. et al., 2024; Wang Y.-T. et al., 2024). Microfluidic reverse electrodialysis systems have been identified because they function as independent biosensing devices which operate without external power sources and provide better mobility for point-of-care diagnostic applications (Yeon et al., 2025). The systems show how microfluidics technology merges with energy-efficient systems to create sophisticated diagnostic instruments that will improve medical testing capabilities during the next few years.

Microfluidic chips enable the study of AD pathogenesis through two new methods: spatial transcriptomics and 3D tissue modeling. The MAGIC-seq platform performs high-throughput transcriptomic analysis of entire tissue sections with near single-cell precision through its microfluidic system, which uses carbodiimide chemistry and spatial combinatorial indexing at affordable costs (Zhu et al., 2024). Mapping the molecular heterogeneity of AD-affected brains and finding new treatment targets are made possible by such skills. Organ-on-a-chip models use patient-derived cells and biomaterials to recreate fibrotic tissue mechanical and biochemical signals to assist in understanding disease origins and developing specific treatments for individual patients (Carvalho et al., 2024; Chen et al., 2025). The new applications have potential to link laboratory studies with medical treatment; however, these systems are still in development.

The detection of AD biomarkers and protein analysis through microfluidic chip technology represents a new approach to solving the problems of sensitivity, accessibility, and scalability that exist in conventional methods. Platforms such as ECL sensors without labels, proteomic multiplex assays, and magnetic nanoparticle concentration systems will transform the field of AD by enabling better early detection and treatment tracking and fundamental research. The development of chips that perform both biomarker detection and drug screening represents a future research direction. The field needs to establish standardized microfluidic methods to enable clinical testing, and machine learning systems must be integrated for data processing (Yu D. et al., 2025). The worldwide fight against AD will receive vital backing from microfluidics technology because scientists will keep advancing this field.

The study of AD biomarkers has received significant advantages from modern microfluidic chip technology, thus enabling better biomarker identification and analysis. These developments make use of the special qualities of microfluidics to improve mobility, multiplexing, and sensitivity, which helps with early detection and comprehension of AD pathophysiology. O’Bryant (2010) highlighted the significance of biomarker profiling in serum samples by demonstrating the potential of serum protein-based multiplex biomarker algorithms for differentiating AD patients from controls. The method shows how microfluidic platforms enable extensive protein detection tests that generate exact results for multiple protein targets simultaneously.

Microfluidics serve as a foundation allowing Yang et al. (2016) to create a quantitative mass spectrometric system to detect proteins at levels that enable individual cell protein analysis. The on-chip iTRAQ labeling system showed how microfluidic technologies enable researchers to perform detailed cellular proteome studies to understand protein changes that occur during AD progression. Li et al. (2020) investigated modern microfluidic system developments to enable biomimetic modeling and AD detection through their ability to process biological materials and nanotechnology-based systems. The new technologies enable functional studies and preclinical tests to be performed directly on their platforms, making biomarker research more adaptable. Mollinari et al. (2022) achieved patient fibroblast conversion into neurons and developed a quick and economical microfluidic method for identifying pathogenic indicators. Their method demonstrates how microfluidic direct conversion methods allow researchers to study neurodegenerative disease biomarkers, which could lead to individualized diagnostic tests.

Jia et al. (2023) created a portable ECL device that uses a bipolar electrode chip to visualize electrochemiluminescence signals for detecting early stages of AD. The system operates at low voltage and follows the current trend of building basic affordable microfluidic devices to perform early detection functions. Gutiérrez-Capitán et al. (2023) created a paper-microfluidic electrochemical device that enables the simultaneous detection of multiple inflammatory biomarkers present in sputum samples. They concluded that paper-based microfluidics work well for medical diagnostic applications.

Although focused on respiratory conditions, the principles of multiplex electrochemical detection are directly applicable to AD biomarker analysis. Lewis et al. (2024) used ACE microfluidic chip technology to detect AD from mild cognitive impairment through plasma extracellular vesicle (EV) biomarkers. EVs function as diagnostic tools to conduct clinical-grade analysis of these vesicles. Li D.-L. et al. (2024) demonstrated a microspectrometer-based microfluidic system that enables real-time protein concentration measurement through a portable diagnostic system with rapid results. These systems hold particular significance for point-of-care applications in AD detection.

In a notable development, Liu et al. (2026) presented MagPEA-POCT, a portable and highly sensitive multiplex platform that employs magnetic-bead-based proximity extension assays. The development of diagnostic systems which can scale up for field use requires this complete system to perform multiplex detection and automated sample preparation for neurodegenerative illness diagnosis. Microfluidic chip technologies have developed to offer portable, multiplexed, and sensitive ways of identifying AD biomarkers. These technologies enable complete proteome and vesicular analysis, which makes early diagnoses easier and has the potential to create personalized medical approaches for neurodegenerative disease treatment.

The flexible design of microfluidic chips enables their use in biomedical applications, including DNA analysis for studying AD genetic factors. The operation of microfluidic devices enables users to perform cell lysis and DNA extraction and amplification and detection procedures that combine to support genetic risk factor identification processes (Bruijns et al., 2016). Moreover, the high-throughput screening capabilities of microfluidic systems are advantageous for drug discovery and mechanistic studies related to AD therapeutics (Cui and Wang, 2019). The combination of microfluidic chip technology with AD research provides new methods to detect diseases at an early stage, create models of the disease, and develop new treatments. The ongoing development of microfluidic systems together with their application methods will establish new possibilities of transforming neurodegenerative disease research and medical treatment practices (Mark et al., 2010; Pattanayak et al., 2021).

Notwithstanding the progress made, substantial challenges remain in standardizing organ-on-chip platforms for clinical translation. It will require complete validation because cell sourcing methods and matrix materials and flow patterns show significant differences between each other. The new method of SiO2-coating PDMS chips serves as a surface protection system that prevents samples from sticking to surfaces, while MAGIC-seq technology with combinatorial indexing enables faster spatial transcriptomics analysis with lower experimental mistakes. Different biological datasets need to be merged with medical models to match each patient to create AD individualized treatments.

### 6.2 Validation of new drugs and repurposing old drugs

The complex nature of AD requires scientists to develop multiple target approaches, which will result in effective treatment solutions. BACE1 (β-secretase 1) inhibitors show promise as treatments because they successfully decrease Aβ generation, identified as the primary pathogenic mechanism of AD progression (Iqbal et al., 2023; Lin et al., 2024; Sheokand et al., 2024). Fresh BACE1 inhibitors stem from marine phytochemicals, fungal metabolites, and plant-derived compounds. Bisacremine-C together with additional fungal compounds showed 25-times better BACE1 binding than their first screening compound, while the plant compounds ponciretin danthron and chrysophanol bound to BACE1 at nanomolar concentrations. Therefore, natural compounds have the ability to function as potent BACE1 blocking agents (Iqbal et al., 2023; Amini et al., 2024; Sangeet and Khan, 2025). The compounds lactodehydrothyrsiferol from red algae and bisacremine-C from fungal metabolites have demonstrated nanomolar strength against BACE1 through their ability to form stable bonds with the protein, validated through molecular dynamics simulations (Iqbal et al., 2023; Sheokand et al., 2024). Natural products serve as important sources of BACE1 inhibitors that show beneficial drug characteristics and safe profiles.

Beyond amyloidogenesis, neuroinflammation and mitochondrial dysfunction are important factors in the development of AD. The process of neuroinflammation develops through reactive astrocyte and microglial activation, leading to increased neuronal damage because these cells produce pro-inflammatory cytokines and oxidative stress (Chacón-Quintero et al., 2021; Sun L. et al., 2024). BACE1 dysregulation in astrocytes causes NVU disruption, which proves that the enzyme plays a role in neuroinflammatory responses (Chacón-Quintero et al., 2021). EGb 761 demonstrates neuroinflammation treatment ability through its ability to manage microglial activation and its capacity to reduce oxidative stress, which protects cognitive function (Sun L. et al., 2024).

The development of new screening methods to combine pharmacophore modeling with quantitative structure–activity relationship (QSAR) analyses enables scientists to find neuroinflammation modulators and mitochondrial protectants more rapidly (Kumar et al., 2020; Bampatsias et al., 2022). E-pharmacophore models have identified ZINC39592220 as a potent BACE1 inhibitor that exhibits antioxidant activity and demonstrates an ability to treat two different medical conditions (Chidambaram, 2023). The QSAR models show which structural components of BACE1 inhibitors include hydrophobic aromatic rings and hydrogen bond acceptors that developers can use to design new BACE1 inhibitor compounds (Kumar et al., 2020). Computational methods establish an effective system to discover multi-target compounds to target BACE1, neuroinflammation, and mitochondrial pathways through high-throughput screening of natural product libraries (Kumar et al., 2020; Chidambaram, 2023; Iqbal et al., 2023).

The therapeutic approach becomes more effective through the implementation of biomarker research as an addition to the treatment protocol. BACE1 enzyme activity in blood serum enables the determination of both the severity of AD progression and the time until patients develop noticeable symptoms, thus showing potential as a diagnostic and predictive tool (Nicsanu et al., 2022). BACE1-AS long noncoding RNA regulates BACE1 gene expression, leading to atherosclerotic cardiovascular disease development and vascular dysfunction (Bampatsias et al., 2022). Medical treatments should use personalized approaches that depend on specific biomarkers to develop customized treatment strategies (Bampatsias et al., 2022; Nicsanu et al., 2022).

A new method is being used to treat AD which combines BACE1 inhibition with treatments for neuroinflammation and mitochondrial protection. The development of biomarker-based treatment methods enables earlier interventions, while using personalized medical approaches and natural products together with computational drug design enables researchers to create new drugs that target multiple disease-related targets. Research is needed to confirm these drugs through laboratory tests and human clinical studies to establish their medical applications.

### 6.3 Patient-specific “avatar” models

Disease mechanisms and drug response tests can be investigated by creating patient-specific “avatar” models developed from fAD and sAD organoid cells. These models employ iPSC technology to generate 3D brain organoids to maintain the individual genetic characteristics of patients while also developing essential AD features, including Aβ plaques, neurofibrillary tangles, and synaptic dysfunction (Mazzocchi et al., 2018; Derouet et al., 2020).

Cancer treatment uses patient-derived 3D organoids to perform personalized therapeutic screening to function as models for studies. Mazzocchi et al. (2018) showed that micro-engineered tumor organoids made directly from fresh biopsies function as individualized models. This assists in finding better treatment approaches before starting new therapies. Similarly, Derouet et al. (2020) established standardized methods to create organoids from esophageal adenocarcinoma biopsy samples to maintain the original tumor conditions before treatment to help develop individualized induction therapy approaches.

Researchers have applied this method to investigate neurodegenerative diseases through human brain organoid models of sporadic AD. Chen X. et al. (2021) employed serum-exposed 3D brain organoids made from human iPSCs to investigate sporadic AD while developing a platform to analyze disease processes and create therapeutic approaches. Ji et al. (2025) developed complex vascularized neuroimmune organoid models that contain all AD-related cell types including neurons, microglia, astrocytes, and blood vessels. Their model showed that brain tissue samples from sporadic AD patients would develop multiple AD-related conditions during 4 weeks of exposure, thus making it suitable for disease research and drug development. The proposed method for enhancing research-to-clinical practice translation uses patient medical records together with organoid models. The research by Dolciotti et al. (2025) showed that the combination of biological organoid models with digital technologies creates new opportunities to improve both disease prediction and prevention in AD research. Integrated methods are being utilized to develop exact disease progression models for humans that overcome the restrictions of conventional medical tests that only identify diseases at their most severe stage. Furthermore, drug testing needs individualized responses because GSK3β inhibitors create distinct effects that depend on the genetic profile of each patient.

The use of patient-derived organoids represents a superior approach to traditional animal models because the latter do not accurately replicate human AD pathology due to differences between species that affect immune responses and gene expression and disease development (Chen X. et al., 2021; Park et al., 2021; 2023). The biochemical and cellular problems that patients experience also occur in fAD organoids that carry PSEN1 or PSEN2 mutations because these cells produce elevated Aβ42:Aβ40 ratios and display hyperphosphorylated tau (Chen X. et al., 2021; Lavekar et al., 2023). Research has used human serum to expose iPSC-derived organoids to BBB leakage conditions because this condition represents more than 95% of AD cases. The exposure of iPSC-derived organoids to human serum enables researchers to study AD because BBB leakage acts as a known AD risk factor that results in elevated Aβ accumulation, neuronal connection destruction, and astrocyte activation of immune responses (Chen X. et al., 2021).

Machine learning algorithms have been used to study calcium oscillation patterns in organoids that produce specific drug responses that doctors can use to create individualized treatment plans (Strong et al., 2023). The models achieve higher accuracy because scientists analyze CRISPR-Cas9-edited isogenic lines that enable them to understand how particular genetic changes influence disease development and medication responses (Park et al., 2021; Strong et al., 2023). The APOE4/4 genotype, which exists in GABAergic neurons of spheroids that resemble prefrontal cortex tissue, causes irregular calcium synchronization; however, blocking NMDA receptors enables researchers to reverse this effect, thus indicating that medical treatment can be tailored to individual genetic profiles (Strong et al., 2023).

The development of scaffold-free methods together with standardized cell differentiation protocols has solved two major problems that had limited organoid production: it has reduced both the differences between separate batches and the formation of dead tissue in the center. These developments make these models suitable for drug screening at high speeds because they produce spheroids with uniform size and cell composition and fully developed functional properties (Chen X. et al., 2021; Park et al., 2023; Strong et al., 2023). The development of retinal organoids from AD patient iPSCs has created an additional tool that enables researchers to study Aβ and p-Tau accumulation in brain tissue through optical imaging for early disease detection (Lavekar et al., 2023). The systems function at scale in biobanks, which maintain frozen organoids for long research durations and multiple patient group drug evaluation (Chen X. et al., 2021; Means et al., 2025).

The NEUBOrg platform and assembloid systems provide two future research methods that use AI to forecast AD development through laboratory experiments and neural circuit modeling using structured organoid arrangements (Esmail and Danter, 2021). Patient-derived organoid avatars enable AD research to advance through the ability to create personalized medication tests and the explanation of disease mechanisms and their role in accelerating precision therapy development. Their technologies together with multi-omics profiling and clinical data analysis are used to find new biomarkers and treatment methods to improve medical outcomes for patients with various health conditions.

## 7 Limitations and future perspectives

### 7.1 Technological bottlenecks

Current 3D models for AD research face technical challenges—most notably the lack of functional perfusable vasculature in human iPSC-derived brain organoids (Pham et al., 2018). This limitation hinders nutrient delivery and the full recapitulation of cerebrovascular dysfunction. While organoid vascularization has been attempted via endothelial cell coating, organotypic brain slices offer an alternative *ex vivo* platform for studying Aβ-induced vessel formation, though they are not human-based organoids and lack blood flow (Steiner and Humpel, 2023). It remains challenging for any single current model to fully combine human cellular complexity with functional perfusable circulation. Current vascularized human brain organoid (vhBO) systems do not fully replicate the complex relationship between neurons and blood vessels because they lack functional perfusable vessels and a mature BBB. As a result, vhBOs cannot yet be used as a model for functional barrier properties, drug transport, or processes that require physiological flow and shear stress (Kistemaker et al., 2025).

Current microfluidic BBB-neurosphere co-culture models can recapitulate short-term vascular phenotypes, such as Aβ-induced barrier permeability increases and localized vascular Aβ deposition, over 7 days of co-culture. However, these models are not vascularized organoids but are, rather, microphysiological systems with perfusable microvascular networks under pressure-driven flow, enabling rapid emulation of AD-associated neurovascular dysfunction without long differentiation times (Ko et al., 2023; Uzoechi et al., 2024). A 3D microfluidic BBB-on-a-chip model using primary human endothelial cells demonstrates that Aβ1-42 oligomers induce dose-dependent tight junction disruption, increased barrier permeability, and endothelial cell death within 48 h under bidirectional flow. This model, while not a vascularized organoid, effectively recapitulates acute Aβ-induced vascular dysfunction and may be used to study cerebral amyloid angiopathy-like phenotypes (Uzoechi et al., 2024). These models have successfully demonstrated that Aβ40 and Aβ42 peptides block endothelial cell migration and cause abnormal vascular development (Deng et al., 2022). Current vascularized human brain organoids and assembloids can recapitulate short-term vascular phenotypes such as endothelial tight junction formation and barrier function. However, most models rely on exogenous Aβ to induce barrier changes; endogenous vascular Aβ deposition has not been demonstrated. Furthermore, vascularized assembloids have been successfully applied to study tauopathy-related glial and inflammatory deficits, but not Aβ pathology (Cakir et al., 2019; Sun X. et al., 2024). What they cannot yet achieve—and this is the critical bottleneck acknowledged here—is the maintenance of long-term, stable, perfusable blood flow, which is essential for modeling chronic processes such as hypoperfusion-driven neurodegeneration, glymphatic Aβ clearance, and age-dependent vascular stiffening.

Current vascularized organoid and organ-on-chip systems exhibit inherent limitations in modeling AD-related vascular pathologies. Chronic hypoperfusion—a sustained reduction in cerebral blood flow lasting weeks to months—and its long-term cumulative effects remain difficult to recapitulate *in vitro*, largely due to core hypoxia/necrosis issues in organoids and the challenges of extended culture periods. Although microfluidic platforms such as those developed can successfully simulate interstitial fluid dynamics and solute clearance over hours to days via hydrostatic pressure gradients, the physiological driving force of arterial pulsatility has not yet been adequately reconstructed, representing a key direction for future chip design. Notably, the p-tau pathology modeled by Park et al. (2025) employs a sub-cytotoxic concentration (1 μM) and primarily induces astrocyte-driven vasoconstriction and impaired glymphatic solute clearance rather than widespread neuronal death. As such, this model is well-suited for investigating early-stage AD events, such as prodromal chronic hypoperfusion and glymphatic dysfunction, rather than late-stage neurodegeneration. Importantly, the study reveals that p-tau promotes astrocytic hypercontractility via the upregulation of phosphorylated myosin light chain II (pMLC2), leading to vessel narrowing and clearance deficits, both of which are reversible by the non-muscle myosin II inhibitor blebbistatin. These findings establish astrocytic contractility as a novel mechano-therapeutic target for restoring glymphatic function in early AD (Larriba-González et al., 2025; Park et al., 2025). While Wörsdörfer et al. (2019) successfully generated vascularized neural organoids that can be maintained for up to 280 days and exhibit hallmarks of vessel maturation—including a basement membrane, pericyte coverage, endothelial junctions, and even microglial-like cell infiltration—their model lacks key biomechanical cues necessary for full vascular maturation. They explicitly note that pulsatile blood-flow-induced shear stress is required for the final differentiation and stabilization of the vessel wall and that this condition is not yet achieved in their static *in vitro* system. Consequently, age-dependent vascular remodeling processes that typically evolve over months to years under continuous hemodynamic forces cannot be faithfully recapitulated in the current setup. Future integration with perfused microfluidic devices or *in vivo* transplantation is proposed to overcome this limitation. Long-term testing of drugs aimed at chronic vascular protection, particularly those requiring assessment of functional vascular responses, is difficult to perform using the organotypic brain slice model, according to Steiner and Humpel (2023). They explicitly note that vessels in these slices are non-functional due to the absence of blood flow, eliminating the possibility of evaluating flow-dependent parameters such as shear-stress-mediated endothelial adaptation or neurovascular coupling. Moreover, while the tissue can be cultured for up to 8 weeks, the collagen-based microcontact prints degrade significantly beyond 14 days, leading to the uncontrolled release of loaded molecules and loss of mechanical guidance. Thus, although the culture window *per se* may accommodate several weeks, the lack of functional perfusion and the limited stability of the hydrogel scaffold preclude any reliable assessment of drug efficacy on vascular remodeling processes that require months of exposure under hemodynamic conditions.

The transplantation of human cortical organoids into the developing somatosensory cortex of immunodeficient neonatal rats represents an effective strategy for achieving functional vascularization and long-term graft survival (Revah et al., 2022). Host-derived endothelial cells and microglia invade the transplanted organoids, as shown by RECA-1 and IBA1 staining, thereby overcoming the perfusable vascular supply that is absent in conventional *in vitro* organoid cultures. T-hCO neurons exhibit significantly advanced maturation across morphological, electrophysiological, and transcriptomic levels compared to stage-matched hCO *in vitro*, including increased soma size, dendritic arborization, spine density, hyperpolarized resting-membrane potential, higher maximal firing rates, and the upregulation of activity-dependent genes. Importantly, t-hCO receives functional thalamocortical and corticocortical inputs from the host brain and extend axonal projections to multiple rat brain regions. Optogenetic activation of t-hCO neurons drives reward-seeking behavior in operant conditioning paradigms, and sensory whisker stimulation evokes calcium transients and spiking responses in human cells. However, this *in vivo* transplantation approach has practical limitations, including the requirement for immunodeficient hosts, sophisticated surgical procedures, and the inability to easily manipulate or monitor the graft at high throughput. Moreover, cross-species temporospatial mismatches between human and rodent development may limit the fidelity of certain circuit-level phenomena.

Practical limitations include the need for highly specialized skills to perform stereotaxic transplantation, inconsistent graft survival rates, the inability to monitor internal graft structures in real time without invasive procedures, and persistent host immune responses that can confound results even in immunodeficient strains. Ethical limitations arise from the use of animal models in chronic AD research that involves human cerebral organoid transplantation (Lavazza and Chinaia, 2023). Key concerns include the welfare of chimeric animals, particularly if transplanted human neural tissue enhances cognitive or sensory capacities to a degree that raises questions about their moral status. Hoppe et al. (2023) propose a hierarchical framework for enhancement and suggest that once certain enhancements are detected, stricter guidelines may be needed to minimize suffering, potentially including enriched housing or retirement to sanctuaries. The threshold at which the functional integration of human neural tissue into host sensory or motor circuits confers morally relevant capacities remains ethically undefined, and current oversight does not provide clear-cut rules.

*Feasibility for AD modeling*. Although transplantation enables vascularization, the procedure is low-throughput and ill-suited for drug screening, which typically requires hundreds of replicates. Moreover, the host vasculature is of rodent origin and may not fully recapitulate human-specific endothelial–Aβ interactions owing to species differences in LRP1 and RAGE expression.

To overcome these bottlenecks, future research should prioritize two-photon polymerization (TPP) printing and microfluidic chip-based approaches that create perfusable, lumenized vascular networks capable of withstanding hemodynamic conditions within organoids. Combining iPSC-derived mural cells (pericytes and smooth muscle cells) with astrocytes can improve NVU network development. CRISPR gene editing can be used to generate isogenic AD models with defined vascular risk alleles to study vessel-specific responses to Aβ accumulation. The combination of these techniques with advanced live imaging will enable the real-time investigation of microvascular blood flow patterns, bridging *in vitro* organoid studies and *in vivo* clinical observations.

#### 7.1.1 Reproducibility, standardization, and critical limitations

Beyond the vascularization bottleneck discussed above, brain organoid technology faces three interconnected challenges that critically affect the reliability and translational relevance of AD modeling: batch-to-batch transcriptomic variability, the confounding influence of iPSC line idiosyncrasies, and methodological heterogeneity in phenotype quantification.

##### 7.1.1.1 Batch-to-batch transcriptomic variability

Even when using the same human iPSC line and standardized differentiation protocols, Chiaradia et al. (2023) demonstrate that cerebral organoids exhibit substantial morphological and transcriptomic variability both across independent batches and within a single batch. By systematically screening protocol variations, they show that organoid morphology—specifically surface complexity, ventricle number and size, and tissue architecture—strongly correlates with transcriptional similarity to the human fetal brain. Organoids with more complex morphology (high-score morphology, HSM) consistently outperform those with simplified morphology (LSM) in matching *in vivo* datasets, regardless of the specific protocol used. Importantly, this relationship is not merely correlative: direct perturbation of tissue architecture leads to aberrant temporal progression, generating cells with mixed progenitor-neuronal identity and disrupted developmental trajectories. Thus, while batch-to-batch variability is an acknowledged limitation, Chiaradia et al. (2023) emphasize that tissue morphology and cytoarchitecture are key determinants of transcriptional fidelity and proper temporal cell fate progression—a finding that cannot be reduced solely to stochastic fluctuations in early neural induction or extracellular matrix composition.

##### 7.1.1.2 The importance of isogenic controls

A critical but subtle issue highlighted by Rouhani et al. (2022) is the necessity of using multiple isogenic controls to distinguish genuine genotype-driven phenotypes from idiosyncrasies of individual iPSC lines. They demonstrate that even when comparing isogenic trisomic and disomic subclones derived from the same parental line, a single-line comparison yields thousands of false-positive differentially expressed non-chr21 genes, whereas an expanded design with three trisomic and three disomic lines reveals no significant non-chr21 differences, despite robust detection of the expected ∼1.5-fold upregulation of chr21 genes. This discrepancy arises because different iPSC clones, even those derived from the same individual and reprogramming experiment, can acquire vastly different somatic mutation burdens due to (i) the oligoclonality of the starting fibroblast population (different subclones carry different UV-damage loads from variable sunlight exposure), and (ii) positive selection for certain mutations that arise during *in vitro* culture and can alter differentiation capacity. Therefore, baseline differences in differentiation efficiency, gene expression, and spontaneous pathology between iPSC lines derived from different individuals, or even from the same individual, are primarily driven by somatic mutations and clonal heterogeneity acquired prior to and during reprogramming rather than by the intended genetic modification. Comprehensive genomic characterization and the use of multiple independent isogenic clones are essential to avoid conflating line-specific idiosyncrasies with genuine experimental effects. Without isogenic controls, observed differences between patient-derived and control organoids may reflect line-specific artifacts (e.g., residual genetic or epigenetic variability) rather than true disease mechanisms. For AD research, this is particularly critical: studies comparing APOE ε4 and APOE ε3 organoids derived from different donors run the risk of confounding genotype effects with other genetic background differences. Both Qu et al. (2024), Park et al. (2021) explicitly address this issue by employing CRISPR-Cas9-edited isogenic lines. Park et al. (2021) generated isogenic ApoE ε4 and ε3 lines from a single parental iPSC line to isolate the effect of the ε4 allele, demonstrating that non-isogenic comparisons can be confounded by other genetic variants. Similarly, Qu et al. (2024) used CRISPR-Cas9 to introduce the APP^N717I^ mutation into a well-characterized control iPSC line, producing multiple independent isogenic clones. Thus, the preferred and validated approach is to generate isogenic series by correcting or introducing the mutation of interest in a single well-characterized iPSC line, combined with the use of multiple independent clones to account for clonal variability. In Gonzalez et al. (2018), cerebral organoids derived from familial AD and DS iPSCs spontaneously developed extracellular Aβ deposits (resembling senile plaques) and intraneuronal phosphorylated tau aggregates (resembling neurofibrillary tangles), as evidenced by immunohistochemistry, thioflavin analog BTA-1 staining, and Gallyas silver staining. To quantify pathological protein accumulation, they employed a fractionation procedure that distinguishes soluble from insoluble aggregates: organoid homogenates were subjected to ultracentrifugation, followed by formic acid extraction of the pellet, allowing ELISA measurements of Aβ40, Aβ42, total tau, and phosphorylated tau (p-tau231, p-tau181, and p-tau396) separately in soluble and insoluble fractions. This approach overcomes the common limitation of ELISA being unable to distinguish oligomers from fibrils, as the insoluble fraction specifically captures aggregated species. Furthermore, spatial information on plaque and tangle distribution was obtained through immunostaining and confocal microscopy, not from ELISA. Thus, while method heterogeneity remains a broader challenge in the organoid field, this study exemplifies a multi-pronged strategy—combining biochemical fractionation with quantitative ELISA and spatially resolved immunohistochemistry—to comprehensively characterize AD-like pathology in human cerebral organoids. Tau pathology was assessed by Bowles et al. (2021) using multiple phospho-epitope-specific antibodies, including AT8 (p-S202/T205), PHF1 (p-S396/S404), and an antibody against p-Tau S396, allowing the detection of different phosphorylated tau species. They observed that P-tau S396 levels increased significantly in tau-V337M organoids between 4 and 6 months, while AT8/PHF1 immunoreactivity was present at 6 months. However, whether these distinct phospho-epitopes correlate with specific stages of organoid maturation or with differential seeding potential remains unexplored. Additionally, Bowles et al. (2021) quantitatively distinguished 3R and 4R tau isoforms by qRT-PCR, revealing that 4R:3R ratios were low and did not differ consistently between mutant and isogenic controls. They acknowledge that the low expression of 4R tau—a hallmark of adult tauopathies—is a limitation of the organoid model. Thus, while they went beyond “total p-Tau” measurements and included isoform analysis, the field still lacks systematic investigations into how specific phospho-epitopes and isoform ratios evolve with organoid maturation and influence tau seeding.

##### 7.1.1.3 Toward standardization

Addressing these limitations requires community-wide efforts to establish minimal reporting guidelines for organoid experiments, including: (i) detailed documentation of differentiation batch number and inter-batch variability metrics; (ii) mandatory use of isogenic controls for genotype–phenotype studies; (iii) orthogonal validation of key findings using at least two independent quantification methods; (iv) deposition of raw data in public repositories to enable meta-analyses. Initiatives such as the Organoid Cell Atlas and the International Society for Stem Cell Research (ISSCR) guidelines provide starting points, but AD-specific standardization remains urgent.

### 7.2 Ethical and social issues

Organoids used in AD research generate various ethical and social problems, which show the sophisticated features of this technology together with its core moral dilemmas. Brain organoids made from human PSCs serve scientists studying AD development through controlled laboratory tests, which produce better results than traditional 2D cell cultures and animal model research. Organoids contain essential features, including tau protein tangles and Aβ plaques, and show better physiological characteristics (Kim et al., 2024). There is ongoing discussion regarding cell brain structure development due to the need to determine whether cells possess consciousness and moral value and what ethical boundaries should exist for research. These cells show brain development patterns that match human development because organoids produce electroencephalogram signals that resemble those of pre-term infants (Hoppe et al., 2023).

Scientists face a major ethical dilemma because they can now maintain cell cultures for extended periods while achieving high functional complexity in AD organoids, which could potentially develop basic forms of consciousness or sentience. Upcoming models will merge sensory data with chimeric integration systems and create new challenges for defining these limits, although current agreements confirm that organoids do not possess sufficient complexity to experience pain or consciousness (Hoppe et al., 2023; Mencattini et al., 2024). Organoids develop at high speed because they reach myelination levels that match 20-week fetal development at 10 weeks of culture time, which raises questions about their potential to experience developmental distress similar to that of early fetal development (Mencattini et al., 2024). AD research has become more complex because organoids used for neurodegenerative disease studies demonstrate neuronal death and dysfunction, which requires scientists to reduce animal distress during experiments (Kim et al., 2024).

The process of obtaining informed consent from biospecimen donors creates a significant obstacle because researchers need to handle organoids from APOE ε and familial AD genetic risk patients with particular care. The donor interview responses indicate their backing for organoids in research, but they want to maintain their involvement to defend ethical principles (MacDuffie et al., 2023). The drug testing capabilities of AD organoids create a conflict between business interests and the rights of tissue donors to decide what happens to their tissue and to receive proper compensation. The present situation requires an immediate solution (Ding et al., 2022; Hoppe et al., 2023). The public disclosure of organoid capabilities through “mini-brains” terminology creates a risk of misleading stakeholders while making social concerns about these technologies more severe (Lavazza and Chinaia, 2023).

There are AD-specific challenges in obtaining truly informed consent from cognitively impaired donors. A distinctive ethical problem in AD organoid research is that the very individuals whose cells are most valuable—patients with familial AD (fAD) or sporadic AD—often have diminished decision-making capacity due to cognitive impairment. This creates a fundamental tension between the ethical imperative of informed consent and the scientific need for disease-relevant biospecimens.

Cognitive deficits have a direct impact on consent capacity in AD. Using the MacArthur Competence Assessment Tool for Treatment (MacCAT-T) in a cross-sectional study of 102 mild-to-moderate AD patients, Lacerda and colleagues found that the four legally recognized abilities for valid consent—understanding, appreciation, reasoning, and expression of choice—exhibited non-linear impairment patterns (Santos et al., 2022). Understanding the deficits correlated with impaired disease awareness, lower self-reported quality of life (QoL), and poorer spoken language comprehension. Appreciation deficits were associated with poorer orientation and older age. Reasoning deficits were associated with poorer orientation and lower self-reported QoL. Expression of choice deficits were solely associated with lower self-reported QoL. Critically, each ability is related to distinct cognitive and clinical deficits, demonstrating that global cognitive functioning alone is insufficient to determine an individual’s decision-making capacity. Fluctuating cognition adds further complexity to assessing decision-making capacity (DMC) in dementia. Cognitive fluctuations—spontaneous alterations in attention, arousal, and cognitive performance—are a diagnostic core feature of dementia with Lewy bodies but also occur in AD and Parkinson’s disease. These fluctuations mean that a patient’s DMC may vary from day to day or even from moment to moment, complicating capacity assessments performed by clinicians or researchers who have only intermittent contact with the individual. Trachsel et al. (2015) emphasize that DMC must be assessed in a case-, task-, and time-specific manner. They recommend that physicians (i) select a moment when the patient is in “good shape”, (ii) improve the patient’s condition if possible, (iii) use simplified information and aids, and (iv) rely only on information obtained during the patient’s best cognitive moments within an assessment session. In contrast to a single evaluation, these strategies implicitly call for repeated or temporally sensitive assessments. However, Trachsel et al. (2015) do not specifically advocate the “supported decision-making model”; instead, they emphasize relational aspects and environmental modifications to foster capacity. Under this model, the supporter provides information and facilitates communication but does not substitute their judgment for that of the patient. This approach enables autonomous participation even when capacity is partially compromised, while safeguarding against undue influence.

When decision-making capacity is substantially impaired (as indicated by an activated Healthcare Power of Attorney), surrogate consent from a legally authorized representative (LAR) is required for research participation. Holden et al. (2018) developed a procedural framework specifically for hospital-based dementia research that uses rapid identification of surrogates—typically the HCPOA—to provide surrogate consent for patients lacking legal capacity. This framework is combined with individualized formal assent procedures for patients who cannot provide full consent. Critically, even when surrogate consent is obtained, individualized assent should be sought from the patient whenever possible respecting residual autonomy; this includes tailored communication based on cognitive ability and observation of behavioral cues for passive assent or dissent. This framework was demonstrated to be effective in facilitating hospital-based recruitment in an ongoing randomized controlled trial (the C-TraC intervention) and provides a basis for increasing access to clinical research for persons with AD and related dementias.

Santos et al. (2022) demonstrated that decision-making capacity in mild-to-moderate AD is domain-specific and cannot be inferred from global cognition alone. However, they did not address iPSC donation or the timing of consent for biological sample collection. Extrapolating these findings to the context of iPSC donation would require additional evidence specific to that decision context.

Brain organoid research is inherently longitudinal—iPSC lines derived from a single biospecimen can be maintained and used for many years or even decades after initial donation. MacDuffie et al. (2023) interviewed 67 biospecimen donors (or parental surrogates) for brain organoid research and found that donors express a strong desire for ongoing engagement with research teams, including (1) learning the results of the research, (2) allowing transfer of decision-making authority over time, and (3) ensuring that ethical boundaries are not crossed. Dynamic consent models—in which donors or their surrogates can revisit and update consent preferences over time as the research progresses—are particularly well-suited to addressing these donor preferences. While MacDuffie et al. (2023) did not focus specifically on AD, their findings are broadly applicable to all brain organoid research, including AD, given the shared need for longitudinal donor engagement and respect for evolving donor values regarding transparency and accessibility of consent information. Informed consent forms for AD patients must be presented in simplified, accessible language that accounts for deficits in understanding and comprehension of spoken language—both of which are impaired even in mild AD.

*Practical recommendations for AD organoid research*. Based on the above analysis, we recommend the following ethical practices for AD organoid studies. (i) Approach potential donors at early disease stages when possible, to obtain truly informed consent before significant cognitive decline. (ii) Conduct serial assessments of decision-making capacity rather than relying on a single evaluation, to account for cognitive fluctuations. (iii) Implement supported decision-making with a trusted advisor. (iv) When capacity is substantially impaired, obtain surrogate consent from a legally authorized representative combined with individualized patient assent. (v) Adopt dynamic consent models that allow donors or surrogates to revisit and update preferences over time. (vi) Present consent information in simplified, accessible language with an explicit disclosure of long-term biospecimen governance. Collaborative efforts between researchers, clinicians, institutional review boards, and patient advocacy groups are essential to developing standardized, ethically-robust consent protocols that are specifically tailored to AD organoid research.

Organoid technology has outpaced the development of robust ethical and legal systems that were intended to govern its use. The current ethical guidelines for human biomaterial research do not solve particular problems that organoids create because they lack definitions about their ethical worth and do not set limits for their use in essential fields such as neuroenhancement and chimera research (Kataoka et al., 2023). Organoid research from particular regions into AD circuit breakdowns has introduced new ethical problems because they create hybrid systems that develop new properties (Hoppe et al., 2023). The current system faces three major obstacles: ownership rights, patent regulations, and worldwide standardization (Kataoka et al., 2023).

AD organoids are a paradigm leap in disease modeling, despite these obstacles. The direct lineage reprogramming of patient-derived fibroblasts produces aged organoids that show reduced heterogeneity, thus creating an exact system for studying APOE ε4-related pathological processes (Kim et al., 2024). Multiple disciplinary review boards need to conduct immediate ethical assessments of this research to determine brain function limits and develop enhanced methods for obtaining organ donation consent from donors (Lavazza and Chinaia, 2023; Mencattini et al., 2024). The evaluation of moral standing through qualitative standards to measure learning ability and stimulus response would give researchers vital direction (Mencattini et al., 2024). The public needs to understand organoid research through engagement programs because scientists need to establish social connections with their audience while making their work widely available (Ding et al., 2022; Lavazza and Chinaia, 2023).

The development of AD organoids requires a complete method which will reveal their full potential while reducing their adverse consequences. The development of organoid models requires scientists to maintain ongoing dialogue with ethicists, legislators, and donors because these models need bioengineering expertise, single-cell genomics, and patient-specific data analysis. The future development of AD organoid research requires the worldwide standardization of protocols, open communication networks, and protective systems against exploitation to achieve ongoing progress.

### 7.3 Future directions

Organoids will become the main research tool for AD studies because scientists will use new techniques to improve their knowledge of the disease. Organoids in AD research have the ability to revolutionize our understanding of neurodegeneration and medication development and personalized treatment approaches. A physiologically realistic platform to simulate AD pathology is provided by 3D brain organoids, including tau tangles and Aβ plaques, which are hallmarks of the illness, since they replicate important characteristics of human brain architecture and function (Esmail and Danter, 2021; Kim et al., 2024). These cells face two main barriers to medical use: their cell development takes too long and their production batches show irregular results, and their aging process remains unfinished (Esmail and Danter, 2021; Kim et al., 2024). By permitting the effective creation of patient-specific organoids with decreased variability, advances in direct lineage reprogramming and bioengineering are overcoming these constraints and improving repeatability and robustness for disease modeling (Esmail and Danter, 2021; Kim et al., 2024). Brain organoids containing the APOE ε4 variant develop AD symptoms more quickly than usual while their gene expression patterns match what scientists find in AD brains, which allows them to study genetic AD susceptibility (Esmail and Danter, 2021; Kim et al., 2024).

AD is a complicated neurodegenerative condition that affects several organ systems in addition to the brain. The current state of research requires new frameworks which explain how brain functions interact with peripheral systems during AD development according to recent computer modeling and systems biology findings. Induced pluripotent stem-cell-derived brain organoids have been developed to study the progressive nature of AD. Researchers have failed to achieve vascularization and multi-organ interactions, which demonstrates the need for computational solutions like NEUBOrg platform that employ machine learning to simulate complete brain operations and body-wide consequences (Esmail and Danter, 2021). Biomarker changes in CSF and neuroimaging results track body-wide changes that include inflammation and metabolic problems through event-based modeling, and thus show promise for AD progression evaluation (Wittens et al., 2025).

The dorsal visual pathway, which includes the V1, V2, and V5 cortical areas, functions as a fundamental system for researchers to investigate how AD causes connections to disappear between brain areas. Neural mass models of this pathway show how brain area connections that are far apart become weaker, resulting in coordination problems that match the EEG patterns found in AD patients (Yan S. et al., 2023). The computational system matches findings from fMRI studies that show that MCI patients have reduced functional brain connections between their brain areas while their dorsal pathway shows abnormal activation patterns that lead to visuospatial performance problems (Esmail and Danter, 2021; Yan S. et al., 2023). Research has used dynamic causal modeling of magnetoencephalography data to understand how glutamatergic receptor dynamics change in AD, revealing how synaptic failures lead to network disruptions throughout the default mode network (Jafarian et al., 2025). To reduce the risk of AD, early therapies that target organ-specific aging could be informed by such systems-level insights.

The development of complete models to study how AD affects multiple body organs has become achievable because of modern technological progress. The AD classification model achieved 93% accuracy through DSL-GNN, which integrates structural and functional organ connectivity data by using graph-based deep learning methods (Wang Y.-F. et al., 2025). The research benefits from single-cell transcriptomic analyses that identify profibrotic cell types that exist in non-neural tissues. The cells that develop AD disease use identical extracellular matrix pathways to create conditions that lead to the disease (Fan et al., 2021). The execution of these strategies demonstrates how multi-organ modeling systems reveal the entire body mechanisms of AD while creating new therapeutic approaches.

Organoid technology has evolved into a revolutionary new method through its combination with machine learning and AI systems. AI technology allows researchers to analyze high-dimensional organoid data, which enables them to forecast drug reactions and detect disease indicators and enhance their experimental methods (Abdollahi, 2021; Mencattini et al., 2024). Scientists are working on hybrid AI–organoid systems that aim to replicate brain functions and evaluate new treatments, although they have not addressed the ongoing moral dilemmas about organoid consciousness (Mencattini et al., 2024; Birtele et al., 2025). The NEUBOrg simulation model for whole-brain organoids operates as a computational system that helps scientists conduct affordable tests of genetic and pharmacological interventions in their laboratory work (Esmail and Danter, 2021). These *in silico* approaches will direct researchers to perform specific experiments that will help them discover new biomarkers and validate new drugs (Esmail and Danter, 2021; Wang D. et al., 2025).

Research into AD has introduced organoids that scientists now use with modern engineering methods, microfluidic systems, and vascular-mimicking scaffolds. Organoids serve as advanced laboratory models that include brain organoids and assembloids to study AD because they duplicate the structure and function of the human brain, thus enabling the study of AD development through advanced physiological studies (Polini and Moroni, 2021; Birtele et al., 2025). Organoid systems currently have restrictions when it comes to duplicating the active relationship between neural cells and vascular elements, which represents a fundamental element of AD disease progression through BBB breakdown and neurovascular system disconnection (Bulboacă et al., 2020; Ko et al., 2023). This problem requires a solution through new microfluidic technology that creates co-culture systems that unite neuronal organoids with vascular components to develop “neurovascular unit-on-a-chip” models. Scientists have developed compartmentalized microfluidic systems that position self-organized microvascular networks against neurospheres to show AD symptoms. The systems have shown two main pathological characteristics: damage to the BBB and specific areas where β-amyloid deposits form (Ko et al., 2023). The systems duplicate the neurovascular interactions that occur in living tissues while allowing scientists to track disease development and treatment effects in real time, thus connecting laboratory 2D cultures to animal models (Liu et al., 2022; Menikdiwela et al., 2025).

Bioengineering scientists have developed superior AD models through their work on organoid development research. The development of neurofluidic devices through microfluidics now includes Tesla-valve-inspired microtunnels and nanoporous microelectrodes, which enable the reverse engineering of neural networks that contain multiple nodes to create brain region feed-forward connections such as the entorhinal-hippocampal circuit that is susceptible to AD (Hanssen et al., 2024). Researchers have used vascular-mimicking scaffolds, which combine fibrin hydrogels with endothelial cells, pericytes, and astrocytes, to create BBB models for AD studies, allowing them to investigate Aβ movement and neuroinflammatory processes (Bulboacă et al., 2020; Ko et al., 2023). The scaffolds enable perfusable capillary network development operating under shear forces that mimic human body conditions and creates an authentic environment for drug testing (Bulboacă et al., 2020; Yan H. H. N. et al., 2023).

The combination of organoids with AI and automation systems will establish a new method for studying Alzheimer’s disease because it enables scientists to conduct extensive and precise research. AI-based analysis of high-dimensional organoid data can identify weak disease indicators, which can be applied to disease progression prediction and the development of personalized treatments (Polini and Moroni, 2021; Birtele et al., 2025). Deep learning algorithms that receive 3D light-sheet imaging data of organoids can measure organoid morphogenetic changes through subcellular detail to perform extensive phenotyping of AD models (Beghin et al., 2022). The use of CRISPR-engineered organoids containing familial AD mutations enables scientists to create specific genetic models, helping them investigate disease processes and develop new gene editing treatment approaches (Hendriks et al., 2021; Wang D. et al., 2025).

New technology development needs ethical frameworks together with regulatory systems to create new guidelines from their existing rules. Currently, neural organoids show no signs of consciousness, yet their increasing complexity requires ethical guidelines to be established to determine their value and the limits of research (Birtele et al., 2025; Igarashi et al., 2025). The process of clinical application requires three essential elements: standardized protocol implementation, high-capacity drug screening systems, and organoid-based biomarker verification methods (Hofer and Lutolf, 2021; Wang D. et al., 2025). Two main obstacles may prevent organoid systems from achieving their complete potential for disease research and treatment development. Current organoid systems need to address their developmental limitations to achieve their maximum potential for disease research and treatment development (Sun and Zhang, 2024).

Organoid development will benefit from technological advances to unite single-cell analysis with new organoid development methods. Recent reviews of technological advancements and optimization methods for AD and other neurodegenerative diseases support the expanded use of organoids in these conditions. Zhao et al. (2025) assessed major developments in organoid technology that focus on neurodegenerative diseases, showing the need for upcoming technological improvements which will solve present challenges and boost disease modeling precision.

Scientists from different fields must work together for AD organoid research to develop models that accurately show how diseases progress and how patients respond to treatments. Organoids will achieve two goals through their solution of present-day restrictions while using new technological advancements, which will advance the understanding of AD mechanisms and enable the development of individualized treatments and protective measures.

## 8 Conclusion

Brain organoid technology has revolutionized Alzheimer’s disease (AD) research because it resolves the issues that previous models failed to overcome. Three-dimensional organoids that contain neurons, glia, and vascular elements now duplicate all essential AD markers, including Aβ plaques, tau propagation, neuroinflammation, and blood–brain barrier failure. Research into disease mechanisms and genotype-phenotype correlations has become more useful now that scientists have the ability to create aging symptoms and perform CRISPR gene editing. High-throughput drug screening and the creation of patient-specific “avatar” models for precision medicine are two other applications for organoids. The model involves two main problems: a lack of established operational protocols and blood vessel structures. Organoids will merge with bioengineering, AI, and systems biology to analyze complex information which will generate complete vascularized models with functioning immune systems.

## Glossary

2D: Two-dimensional
3D: Three-dimensional
Aβ: Amyloid-beta
AD: Alzheimer’s disease
ADOD: Alzheimer’s disease and other dementias
AI: Artificial intelligence
ANR: Anterior neural ridge
APOEε4: ε4 allele of the apolipoprotein E gene
APP: Amyloid precursor protein
ASOs: Antisense oligonucleotides
BACE1: β-Secretase 1
BBB: Blood–brain barrier
BMPs: Bone morphogenetic proteins
CAA: Cerebral amyloid angiopathy
CoM: Coffin-like microglia
CSF: Cerebrospinal fluid
DAM: Disease-associated microglia
DMTs: Disease-modifying treatments
EB: Embryoid body
EC: Endothelial cells
ECM: Extracellular matrix
EV: Extracellular vesicle
fAD: Familial Alzheimer’s disease
FGF: Fibroblast growth factor
FTD: Frontotemporal dementia
GSK3β: Glycogen synthase kinase 3β
**H**_**2**_ **O**_**2**_: Hydrogen peroxide
HGPS: Hutchinson–Gilford progeria syndrome
HiDFP: Human induced dorsal forebrain precursor
HR: Homologous recombination
IN: Induced neuron
iPSC: Induced pluripotent stem cell
LMICs: Low- and middle-income countries
MEAs: Microelectrode arrays
MGCs: Microglia-like cells
NVU: Neurovascular unit
PaM: Plaque-associated microglia
PSCs: Pluripotent stem cells
QSAR: Quantitative structure-activity relationship
ROS: Reactive oxygen species
sAD: Sporadic AD
SDI: Socio-demographic index
SERS: Surface-enhanced Raman scattering
SHH: Sonic hedgehog
SIPS: Stress-induced premature senescence
TPP: Two-photon polymerization
UPCP: Unique progerin C-terminal peptide
VO: Vascular organoid
VSMCs: Vascular smooth muscle cells
WNT: Wingless-related integration site
ZLI: Zona limitans intrathalamica
